# Clonality, management and geography shape genetic structure in a perennial species with restricted distribution

**DOI:** 10.1093/aob/mcag132

**Published:** 2026-05-15

**Authors:** Irene Martínez-Velasco, Jaime Gasca-Pineda, Alicia Mastretta-Yanes, Roberta Gargiulo, Anastasia Klimova, Erika Aguirre-Planter, Alfonso Valiente-Banuet, Rafael Lira, Luis E Eguiarte

**Affiliations:** Departamento de Ecología Evolutiva, Instituto de Ecología, Universidad Nacional Autónoma de México, Ciudad de México CP 04510, México; Posgrado en Ciencias Biológicas, Universidad Nacional Autónoma de México, Ciudad de México CP 04510, México; Departamento de Ecología Evolutiva, Instituto de Ecología, Universidad Nacional Autónoma de México, Ciudad de México CP 04510, México; Ecosystem Stewardship, Royal Botanic Gardens, Kew, Richmond, London TW9 3AE, UK; Ecosystem Stewardship, Royal Botanic Gardens, Kew, Richmond, London TW9 3AE, UK; Departamento de Ecología Evolutiva, Instituto de Ecología, Universidad Nacional Autónoma de México, Ciudad de México CP 04510, México; Departamento de Ecología Evolutiva, Instituto de Ecología, Universidad Nacional Autónoma de México, Ciudad de México CP 04510, México; Departamento de Ecología de la Biodiversidad, Instituto de Ecología, Universidad Nacional Autónoma de México, Ciudad de México CP 04510, México; Unidad de Biotecnología y Prototipos (UBIPRO), FES Iztacala, Universidad Nacional Autónoma de México, Tlalnepantla, Estado de México, México CP 54090, México; Departamento de Ecología Evolutiva, Instituto de Ecología, Universidad Nacional Autónoma de México, Ciudad de México CP 04510, México

**Keywords:** *Agave karwinskii*, conservation unit, effective population size, local adaptation, genetic conservation, mezcal, Mexico, partial clonal system, traditional management, restriction site-associated DNA sequencing

## Abstract

**Background and Aims:**

Genetic information is essential for understanding evolutionary processes across different temporal scales, particularly those occurring over contemporary or recent generations influenced by management, and for guiding conservation actions. These components are now explicitly incorporated into the Kunming–Montreal Global Biodiversity Framework (GBF), which emphasizes metrics such as the effective population size as key indicators for monitoring and management. However, the genetic context of partly clonal and economically important systems remains insufficiently explored. *Agave karwinskii*, a mezcal-producing species with a restricted distribution in southern Mexico, which occurs along a gradient of management intensity dominated by clonal propagation, provides an excellent model for informing conservation strategies in these understudied systems.

**Methods:**

We generated genomic data using restriction site-associated DNA sequencing and conducted population-level analyses, integrating the biological and management context of the species to understand its genetic patterns and delineate conservation units. We also evaluated GBF-relevant indicators to assess genetic diversity and support management planning.

**Key Results:**

*Agave karwinskii* exhibited high genetic diversity (*Hs* = 0.25) and moderate population differentiation (*F*_ST_ = 0.19), shaped by geographical, ecological and, particularly, management factors. We identified 173 single nucleotide polymorphisms as candidate loci potentially associated with local adaptation to environmental variation, including 15 robust single nucleotide polymorphisms consistently detected by four complementary approaches and associated with defence responses, adaptation to environmental stressors, growth and metabolic regulation. Two evolutionarily significant units and seven management units were delineated. The effective population size declined sharply in clonal and semi-cultivated systems, with most units falling below the GBF threshold (*N_e_* > 500), despite large census sizes. Traditional mixed management helps to maintain diversity by promoting gene flow and reducing inbreeding, acting as a form of *in situ* conservation.

**Conclusions:**

This study provides a population genetic framework for understanding evolutionary processes influenced by management and guiding conservation strategies in *A. karwinskii*, offering insights applicable to other perennial, partly clonal and semi-domesticated plant species.

## INTRODUCTION

Genetic diversity plays a crucial role in the ability of species to face current and future biotic and abiotic threats (Hoban *et al.*, 2020). In this sense, describing and understanding genetic diversity is central to inferring evolutionary processes operating over both recent and long-term time scales in any species, and for preserving biodiversity itself. Indeed, genetic diversity is now a formal target within the Kunming–Montreal Global Biodiversity Framework (GBF).

In this context, contemporary effective population size (*N*_e_) is a central indicator because it reflects the rate at which genetic diversity is lost through genetic drift (Frankham *et al.*, 2014; Hoban *et al.*, 2024). In the absence of genetic data, *N*_e_ can be inferred from census size (*N*_c_, the raw count of mature individuals), although this relationship is highly context dependent, and it is influenced by life-history traits, variance in reproductive success and, in dioecious species, the breeding sex ratio (Frankham *et al.*, 2014; Hoban *et al.*, 2024). This is particularly evident in clonal plants, where estimation of *N*_e_ using this approach is challenging because *N*_c_ conflates sexually produced genets (genetically distinct individuals) with clonally propagated ramets (Gargiulo *et al.*, 2023).

Despite their ecological and economic importance, partly clonal species remain largely absent from current conservation frameworks, including genetic monitoring schemes and genetic diversity indicators (Hoban *et al.*, 2024). In addition, species of economic value are under more pressure, such as the extraction of seeds and adult plants, and artificial selection. These pressures can reduce, maintain or even increase the genetic variation of the populations (Aguirre-Dugua and Eguiarte, 2013; Casas *et al.*, 2024) and *N*_e_ (Frankham *et al.*, 2014). A prime example of this dynamic occurs in Mesoamerica, where both *in situ* and *ex situ* management of wild plants within forests and agroecosystems is common, involving diverse practices that promote the development, care and protection of plants (Casas *et al.*, 2024).

Nevertheless, agricultural systems worldwide, including those in Mesoamerica, have shown a strong trend towards technification and monoculture. This shift is clearly evident in the cultivation of agaves, which are one of the most socially and economically important crops, owing to their role as the raw material for mezcal, a distilled alcoholic beverage with deep roots in Mexican tradition (Zizumbo-Villarreal *et al.*, 2013; Valiente-Banuet, 2023). To maximize sugar content for alcohol production, the inflorescence of mature plants is cut before flowering. However, because agaves reproduce sexually only once in their lifetime (i.e. the display semelparous flowering), this practice suppresses seed formation and eliminates a crucial source of genetic variation. Combined with the suppression of sexual reproduction and the selection of a limited number of high-performing genotypes, these practices have reduced genetic diversity and weakened the adaptive capacity of agaves to cope with environmental change (Valiente-Banuet, 2023), as reported in other studies of these economically important plants [for a comparative synthesis of restriction site-associated DNA sequencing (RADseq) studies across agaves, see Martínez-Velasco *et al.*, 2026]. However, this pattern is not universal, because other species in the genus (for instance, *Agave angustifolia*) can exhibit high levels of genetic variation (Klimova *et al.*, 2022), highlighting the complexity of domestication and management effects on agave genetic diversity.

**Fig. 1.**
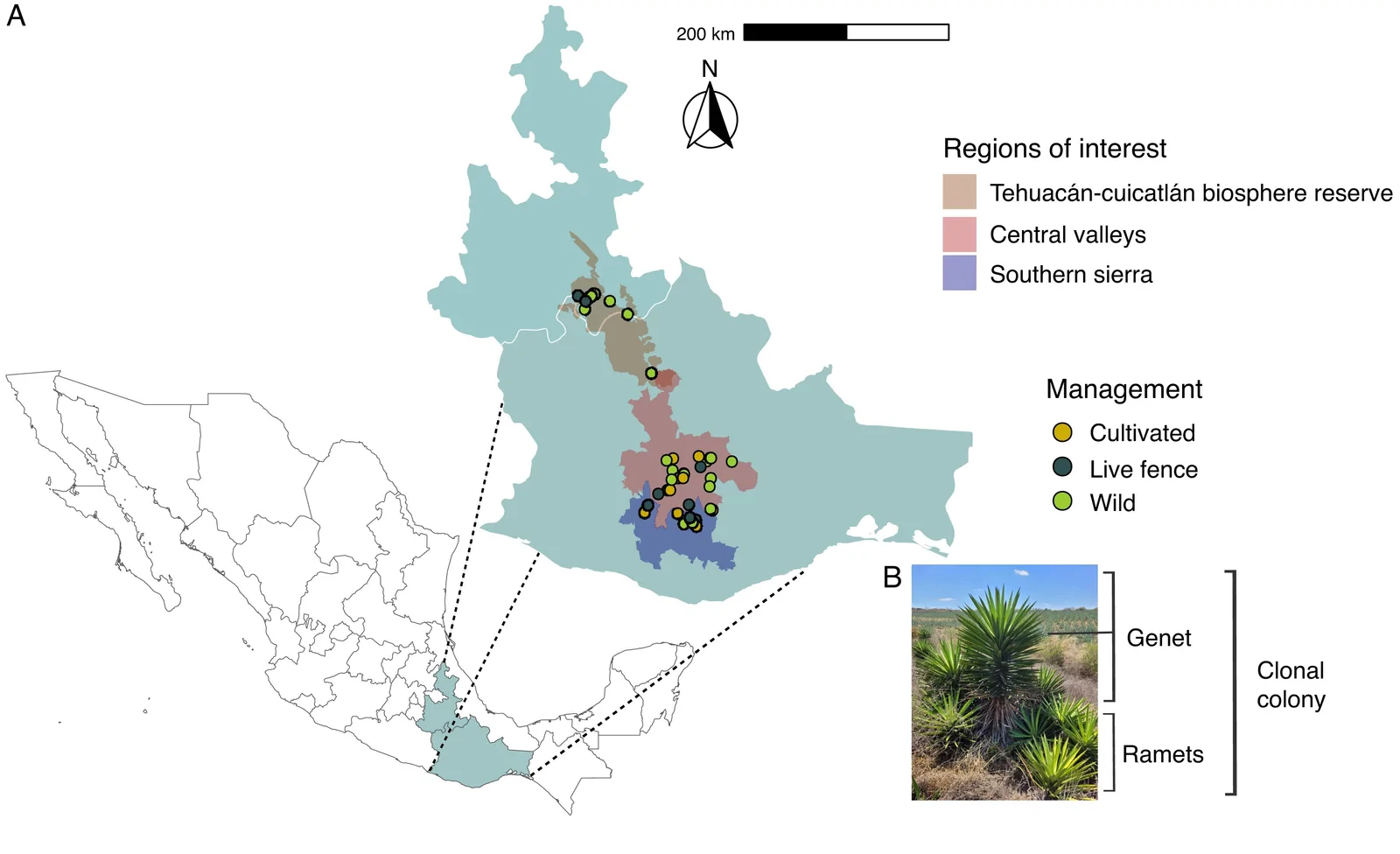
(A). *Agave karwinskii* distribution and sampling sites. In Puebla, populations extend from Chilac to Zapotitlán Salinas within the Tehuacán–Cuicatlán Biosphere Reserve. In Oaxaca, the species occurs in the Central Valleys and in a small portion of the northern Sierra Sur. Sampling localities are shown as dots, with colours indicating the management context of the individuals. (B) *Agave karwinskii* exhibits a partly clonal reproductive system, forming clonal colonies through the production of ramets (genetically identical offshoots or clones) arising from rhizomes, as illustrated in this clonal colony. In this study, only the main ramet was collected, maintaining a minimum distance of 10 m between samples to ensure that each represented a unique genet. Photograph by Irene Martínez-Velasco.

Within mezcal-producing agaves, species with restricted distributions face particular challenges, because they display reduced genetic diversity owing to small, fragmented populations and limited dispersal capacity (Souza *et al.*, 2022). Human-driven habitat fragmentation, land-use change, the loss of pollination corridors and the disruption of trophic networks compound these risks further (Valiente-Banuet, 2023). *Agave karwinskii* Zucc. exemplifies this vulnerability, because it is confined to the Mexican states of Puebla and Oaxaca (Fig. 1A), where its habitat is being lost owing to land-use change associated with the growing mezcal industry (Vázquez-Pérez, 2015; Flores, 2022).

Despite having a relatively small distribution, *A. karwinskii* shows a high morphological diversity of cultivars within the state of Oaxaca, known by different local names and described as ‘ethnotaxa’ in previous research (Vázquez-Pérez, 2015). This diversification has been related to the high plasticity of the species and its historical association with indigenous communities, such as the Mixtecos and Zapotecos (Urrutia-Cruz, 1986; Vázquez-Pérez, 2015). However, this agave has transitioned from being a supplementary resource in mezcal production to becoming a key industrial species, a shift driven largely by the increasing national and international demand for mezcal, which has, in turn, promoted the intensification of its traditional management into agricultural practices.

*Agave karwinskii* is cultivated across a spectrum of contexts: from wild populations (where individuals are either directly harvested or actively promoted through vegetative transplanting both *in situ* and *ex situ*) to agroecosystems intercropped with other species and, more recently, to large-scale agro-industrial plantations. All these systems rely on the partly clonal reproductive strategy of the species, in which the primary mode of propagation is asexual (Fig. 1B). However, increasing demand has accelerated the shift towards clonal propagation, while simultaneously driving the overexploitation of wild plants and seeds, severely limiting natural regeneration (Irene Martinez-Velasco, personal observation). Owing to the combined threats of habitat loss and its increased clonal propagation, the species has been classified as ‘Vulnerable’ by the IUCN Red List (García-Mendoza *et al.*, 2019).

The aim of our study was to evaluate the genetic diversity of *A. karwinskii* to inform its conservation and sustainable management. Our primary objective was to understand the evolutionary and anthropogenic processes shaping its current genetic landscape. To achieve this, we first assessed how different degrees of agricultural management impacted the genetic diversity and population structure of the species, providing a basis for defining management units (MUs), which are demographically independent populations with restricted gene flow among them (Funk *et al.*, 2012). Second, we identified candidate loci showing signatures consistent with local adaptation (Formenti *et al.*, 2022). Integration of both sources of information allowed us to delineate evolutionary significant units (ESUs; Funk *et al.*, 2012). Finally, we addressed the challenge of estimating *N*_e_ in a partly clonal plant, contributing to empirical data to refine *N*_e_/*N*_c_ ratios and facilitate the implementation of the genetic diversity indicators proposed in the GBF (Hoban *et al.*, 2024; Mastretta-Yanes *et al.*, 2024*a*).

## MATERIALS AND METHODS

### Sample collection, DNA extraction and genomic data generation

A total of 300 *A. karwinskii* plants were sampled across the whole natural species distribution range (GBIF.org; inaturalist.org; Vázquez-Pérez, 2015; Fig. 1A). The sampling strategy was designed to maximize the collection of unique genets while avoiding the resampling of clonal ramets (Fig. 1B).

Management categories were assigned based on field observations as follows: sites without human intervention were classified as wild; individuals used as live fence, ornamentals or to demarcate land boundaries (where their growth is tolerated or promoted alongside other crops) were classified as live fences; and individuals propagated vegetatively and grown in monoculture under intensive management were classified as cultivated. A total of 39 sampling sites were included, comprising 17 wild sites (120 individuals), 13 live-fence sites (94 individuals) and 9 cultivated sites (86 individuals) (Fig. 1A; Supplementary Data Table S1). Prior informed consent for sample collection was obtained from all participating mezcal producers and local communities.

Young and fresh leaf tissue (∼10 cm) was collected, stored in paper bags and kept cold in insulated containers with ice packs during transport to the Laboratory of Molecular and Experimental Evolution at the Instituto de Ecología, Universidad Nacional Autónoma de México (UNAM) in Mexico City, where it was stored at −20 °C until DNA extraction. The remaining plant tissue was stored at −80 °C in the same institute.

We extracted DNA from frozen leaves following the modified CTAB ‘Mini-Prep’ protocol (Klimova *et al.*, 2022). DNA quantity was determined using a Qubit 3.0 fluorometer (dsDNA BR Assay Kit, Thermo Fisher Scientific, USA), and quality was verified with a NanoDrop Lite Spectrophotometer (Thermo Fisher Scientific, USA).

RADseq libraries were prepared by the University of Wisconsin–Madison Biotechnology Center (https://biotech.wisc.edu/) following the genotyping-by-sequencing protocol of Elshire *et al.* (2011) with minor modifications, using a double-enzyme digestion (PstI and MspI). A total of 300 samples were sequenced on an Illumina NovaSeq platform (2 × 150 bp).

Adapter sequences and low-quality bases were removed from raw reads using TRIMMOMATIC (Bolger *et al.*, 2014). Paired-end FASTQ reads were demultiplexed, quality filtered and, finally, assembled to an *Agave tequilana* Weber transcriptome (GAHU00000000.1; Gross *et al.*, 2013) using ipyrad v.0.9.79 (Eaton and Overcast, 2020), with recommended parameters for paired-end RADseq (pairddrad).

Subsequent filtering was performed using VCFtools v.0.1.15 (Danecek *et al.*, 2011). Only loci with a minimum and maximum depth of 12 and 200 reads, respectively, and with a maximum of two alleles (excluding InDels) were retained. Consistent results across minor allele frequency (MAF) thresholds (0.02 and 0.05) indicate that rare alleles had a minimal effect on genomic patterns. We therefore used a threshold of 0.05 to balance artefact reduction and biological signal (O’Connor *et al.*, 2015). DNA sites with >20 % missing data were excluded. Single nucleotide polymorphisms (SNPs) deviating significantly from Hardy–Weinberg equilibrium (*P* ≤ 0.05 after Bonferroni correction) were also filtered out. Linkage disequilibrium (LD) pruning was performed using plink v.1.9 (Purcell *et al.*, 2007). Markers were removed based on the previously estimated correlation between each pair of loci (*r*^2^), using a dataset-adjusted threshold of 0.35 (−indep-pairwise 100 10 0.35). For the identification of putatively selected SNPs, Hardy–Weinberg equilibrium filtering was not applied.

The full dataset (300 individuals) was used to assess overall genetic diversity, differentiation and signals potentially associated with local adaptation. Meanwhile, for exploratory purposes and to disentangle the effects of human management, we prepared an additional dataset with only wild samples, used to evaluate natural evolutionary patterns through population structure analyses. VCF files were generated for subsequent analyses, with all previously mentioned filters applied independently to each one.

Clonal structure was resolved using the poppr package (Kamvar *et al.*, 2014) in R (R Core Team, 2024), identifying multilocus genotypes and applying functions such as missingno by removing loci with missing data (cut-off = 1). Informative loci were retained using informloci (see Results), and clonecorrect to exclude redundant genotypes. To account for potential scoring errors or somatic mutations accumulated over the long lifespan of these plants (Denham *et al.*, 2020), multilocus genotypes were grouped further into multilocus lineages using a genetic distance threshold that clustered genotypes derived from the same clonal ancestor (Kamvar *et al.*, 2014).

### Analysis of genetic diversity

Basic population genetic statistics were computed for the entire dataset and by management category. Expected heterozygosity (*H*_e_) and observed heterozygosity (*H*_o_), along with Wright’s inbreeding coefficient (*F*_IS_), were estimated using adegenet v.2.0.1 (Jombart, 2008). Two additional individual-based inbreeding estimators [the *f* coefficient (ratio of observed to expected homozygosity) and *Fhat3* (Yang *et al.*, 2011)] were obtained with plink v.1.9 (Purcell *et al.*, 2007).

Individual heterozygosity was quantified further using inbreedR (Stoffel *et al.*, 2016), calculating multilocus heterozygosity (MLH) and standardized multilocus heterozygosity (sMLH). Differences in diversity and relatedness among management categories were tested using the Wilcoxon rank-sum test implemented in ggpubr (Kassambara, 2026).

Finally, site-wise weighted nucleotide diversity (π) was estimated using pixy (Korunes and Samuk, 2021), assigning weights based on the number of genotyped samples per site. The number of private alleles per group was obtained using the populations module of Stacks v.2.68 (Catchen *et al.*, 2013).

### Genetic differentiation and defining management units

To define MUs, we applied the framework proposed by Funk *et al.* (2012), adapted to incorporate management practices, and reported ethnotaxa as an additional subcategorization criterion.

MUs were delineated using multiple approaches. Initially, a genetic distance matrix among individuals was calculated based on the proportion of distinct loci using the bitwise.dist function in the poppr package (Kamvar *et al.*, 2014). These distances were then visualized in a Unweighted Pair Group Method with Arithmetic Mean (UPGMA) dendrogram with 1000 bootstrap replicates to assess cluster support.

We also conducted a principal component analysis (PCA) and a discriminant analysis of principal components (DAPC) using the adegenet package v.2.0.1 (Jombart, 2008), with interpretation focused on the main axes of variation, which are robust to random noise in high-dimensional datasets (Hellton and Thoresen, 2014). For the DAPC, groups were defined *a priori* based on clustering patterns identified in the UPGMA dendrogram (see above). The number of principal components (PCs) retained was determined using a cross-validation procedure implemented in adegenet (xvalDapc), which identified 60 PCs as optimal based on the lowest mean squared error. The number of discriminant functions retained was selected based on inspection of the eigenvalue bar plot from the discriminant analysis. To further explore fine-scale genetic structure within the main genetic grouping identified in the initial analysis, a nested DAPC was subsequently performed following the same procedure. In this case, the optimal number of retained PCs was again determined by cross-validation (40 PCs).

Additionally, we used a Bayesian clustering approach via STRUCTURE v.2.3 (Pritchard *et al.*, 2000) to infer the most likely number of genetic clusters (*K*). We explored values of *K* ranging from 1 to 15, with 15 independent runs per *K* value, each consisting of a burn-in period of 100 000 iterations followed by 200 000 Markov chain Monte Carlo (MCMC) iterations. Analyses were conducted using the admixture ancestry model with correlated allele frequencies. The most likely number of clusters was evaluated initially using the Δ*K* method (Evanno *et al.*, 2005). However, because Δ*K* primarily detects the uppermost hierarchical level of structure and can underestimate biologically meaningful subdivision, final interpretation of clustering patterns was based on congruence with UPGMA results, in addition to correspondence with ethnobotanical groupings (see Results), whereas lower *K* values supported by Δ*K* are shown in the Supplementary Data Fig. S3.

To investigate recent shared ancestry among individuals, we used an MCMC-based approach implemented in fineRADstructure (Malinsky *et al.*, 2018). We used the RADpainter module to generate a co-ancestry matrix summarizing nearest haplotype relationships across the dataset. The analysis was run for 1 000 000 MCMC iterations with an equal-length burn-in, sampling every 1000 iterations. The final tree was constructed using 100 000 iterations. Results were visualized using the scripts fineradstructureplot.R and finestructurelibrary.R (https://github.com/edgardomortiz/fineRADstructure-tools).

Genetic differentiation between MUs was estimated using pairwise *F*_ST_ values calculated with the pairwise.neifst function from the hierfstat package (Goudet, 2005). Ninety-five per cent confidence intervals (CIs) were obtained using the boot.ppfst function from the same package.

To strengthen inferences about spatial genetic patterns, we integrated genetic and geographical data. Isolation by distance was evaluated using a Mantel test between genetic and geographical distance matrices, implemented via the mantel function in the vegan package v.2.5-7 (Oksanen, 2015). Genetic distances among individuals, previously calculated with bitwise.dist (poppr), and pairwise *F*_ST_ values (hierfstat) were each compared with geographical (in kilometres) and elevational distances computed using the dist function (stats). The strength and significance of the correlations were assessed with 999 permutations, and *P*-values correspond to the proportion of randomized correlations equal to or greater than the observed value.

### Local adaptation analyses

To explore how multivariate environmental factors affect genetic composition across geographical regions, we performed a redundancy analysis (RDA). Genome–environment association methods, such as RDA, aim to identify adaptive loci significantly associated with environmental variables, under the hypothesis that certain alleles might confer a selective advantage in specific environments (Capblancq *et al.*, 2018).

The environmental variable matrix was constructed using data from the BIOCLIM database (Booth *et al.*, 2014) and the SoilGrids 2.0 global soil dataset (Poggio *et al.*, 2021). To reduce multicollinearity among predictors, the pairs.panels function from the *psych* package (Revelle and Revelle, 2023) was used to assess their contribution to the analysis. Ultimately, seven bioclimatic layers and seven edaphic layers were retained (Supplementary Data Table S2).

Therefore, to identify candidate loci with a strong adaptive signal (minimizing the inclusion of false positives) confidently, we sought a consensus among multiple outlier detection methods (Forester *et al.*, 2018) that supplemented our RDA analysis with both differentiation-based analysis and PCA. Initially, we used two methods requiring prior population assignments based on our previously identified MUs. The program BayPass was implemented to detect candidate loci showing signatures consistent with selection, while accounting for shared demographic history among populations through a covariance matrix (Ω) (Gautier, 2015). We estimated the XtX statistic, analogous to *F*_ST_ but corrected for population structure, and considered loci with extreme XtX values as candidates under selection. We also used FSThet (Flanagan *et al.*, 2017), which identifies loci with genetic differentiation levels that deviate from neutral expectations based on heterozygosity (Beaumont and Nichols, 1996). To complement the above analyses, we implemented the multivariate method PCAdapt (Luu *et al.*, 2017), which identifies outliers based on Mahalanobis distance without requiring *a priori* population assignments.

For all outlier detection methods, candidate loci were identified using a stringent false discovery rate threshold of *q* < 0.001 (Benjamini and Hochberg, 1995), applying an additional Bonferroni correction in PCAdapt as the most conservative criterion. Consensus loci were defined as those consistently identified across all methods (RDA, PCAdapt, FSThet and BayPass), thus representing a highly conservative subset of candidate loci.

Functional characterization of consensus loci was carried out in two steps: (1) the consensus SNPs were mapped against the reference transcriptome of *A. tequilana* Weber using the program SeqKit (Shen *et al.*, 2016); and (2) candidate genes associated with these loci were identified using BLASTx (Altschul *et al.*, 1997) against the UniProt database, which provides extensive protein-level information (UniProt Consortium, 2015).

### Estimation of the effective population size

To obtain a robust and nuanced estimate of the contemporary *N*_e_, we used two complementary methods: sibship assignment (SA) and linkage disequilibrium (LD). *N*_e_ was estimated at two hierarchical levels. Initially, individuals were grouped into eight genetic groups corresponding to the distinct populations identified in the genetic differentiation analyses (see Results). Subsequently, a subset of these genetic groups was further subdivided according to management type, resulting in a total of 11 management-defined groups reflecting current anthropogenic selection pressures.

For the LD method, we used NeEstimator v.2.1 (Do *et al.*, 2014). To optimize the precision of our estimates, we initially assessed sensitivity to missing data, applying 20 and 15 % thresholds per locus. Given that the 15 % threshold consistently yielded narrower CIs and resolved infinite bounds, this filter was applied to the final dataset. For allele frequency filtering, our primary approach followed the recommendation of Waples (2024) by screening out only singleton alleles. This choice was validated by preliminary tests, in which stricter MAF thresholds (e.g. 0.02 and 0.05) caused a downward bias and instability in *N*_e_ estimates. For all runs, we assumed random mating and calculated 95 % CIs via the jackknife method (Do *et al.*, 2014; Jones *et al.*, 2016).

For the SA approach, we used Colony2 v.2.0.6.3 (Jones and Wang, 2010), which infers parentage and sibship from multilocus genotypes. To ensure comparability across the SA and LD methods, the same final filtered dataset (15 % missing data) used in NeEstimator was also used for all Colony2 analyses.

Analyses were conducted assuming a polygamous mating system, monoecy, potential inbreeding and the absence of clonality, given that the sampling strategy deliberately excluded clones. The genotyping error rate was set to 0.001 (equivalent to one error per 1000 genotypes), and no prior information on parental genotypes was included. We used the full-likelihood method with medium search effort to identify the most likely sibship configuration.

Populations with fewer than ten individuals yielded unreliable estimates of *N*_e_ and were therefore excluded from the final comparative analysis. Based on this criterion, three populations were excluded from the comparisons of *N*_e_.

## RESULTS

### Genomic data generation

The RADseq of 300 *A. karwinskii* individuals generated a total of 1 231 317 472 raw paired-end reads, with an average of 3 997 989 reads per sample, ranging from 241 488 to 9 084 101. After quality filtering and adapter removal, 92.33 % of high-quality reads were retained. Based on these data, 1 591 653 variants were called using the *A. tequilana* Weber transcriptome (Gross *et al.*, 2013) as a reference. The final dataset, after additional filtering steps, included 7156 SNPs, with 14.7 % missing data and a mean individual sequencing depth of 60.81×.

All multilocus genotypes were unique, and each individual was classified as a distinct multilocus lineage, indicating that all samples represent unique genets and that our SNP dataset had high discriminatory power.

### Analysis of genetic diversity

Analysis of 7156 SNPs revealed similar observed heterozygosity (*H*_o_) and expected heterozygosity (*H*_s_) across wild, cultivated and live-fence sites, with an overall genetic diversity of *H*_T_ = 0.25 (s.d. = 0.01) (Table 1). Wild sites displayed the highest diversity (*H*_o_ = 0.24; s.d. = 0.14), followed by cultivated sites (*H*_o_ = 0.23; s.d. = 0.14) and live-fence sites (*H*_o_ = 0.22; s.d. = 0.12), with significant differences among groups (*P* = 0.05).

**Table 1.** Summary of genetic diversity values for individuals under different levels of management of *Agave karwinskii* in the states of Oaxaca and Puebla.

Parameter	All	Wild	Live fence	Cultivated
*n*	300	120	94	86
*H*_s_	0.25 (0.13)	0.25 (0.13)	0.25 (0.13)	0.25 (0.13)
*H*_o_	0.23 (0.14)	0.24 (0.14)	0.22 (0.12)	0.23 (0.14)
*H*_T_	0.25 (0.13)	0.26 (0.13)	0.25 (0.13)	0.25 (0.13)
MLH	0.22 (0.05)	0.23 (0.06)	0.20 (0.08)	0.22 (0.07)
sMLH	0.94 (0.21)	1.00 (0.26)	0.85 (0.34)	0.93 (0.30)
*F*_IS_	0.08 (0.04)	0.06 (0.18)	0.12 (0.19)	0.07 (0.19)
*f*	0.14 (0.27)	0.08 (0.24)	0.22 (0.30)	0.15 (0.27)
*Fhat3*	0.15 (0.25)	0.09 (0.21)	0.22 (0.29)	0.14 (0.24)
π	0.25 (0.01)	0.25 (0.01)	0.25 (0.01)	0.24 (0.01)

The s.d. is shown in parentheses. Abbreviations: *f*, inbreeding coefficient; *Fhat3* index; *F*_IS_, Wright’s inbreeding coefficient; *H*_o_, observed heterozygosity; *H*_s_, expected heterozygosity; *H*_T_, total heterozygosity; MLH, multilocus heterozygosity; *n*, number of individuals per dataset; sMLH, standardized multilocus heterozygosity; π, standardized nucleotide diversity.

Overall nucleotide diversity was high (π = 0.25, s.d. = 0.01), with only a slight reduction in the cultivated group. No private alleles were detected in association with management type (Table 1).

Wright’s inbreeding coefficient (*F*_IS_) across the full dataset was slightly positive (*F*_IS_ = 0.08, s.d. = 0.04), indicating a mild excess of homozygotes. Although wild and cultivated sites showed similar *F*_IS_ values, the difference was statistically significant (*P* = 2.39 × 10^−3^). Live-fence sites exhibited the highest homozygosity (*F*_IS_ = 0.12, s.d. = 0.19), with highly significant differences in comparison to both wild and cultivated groups (*P* = 2.2 × 10^−16^). Individual-level inbreeding, estimated with the *f* index and *Fhat3*, showed consistent patterns: lowest in wild sites (∼0.08–0.09), intermediate in cultivated sites (∼0.14–0.15) and highest in live-fence sites, more than double that of wild sites (*f* = 0.14, s.d. = 0.27; *Fhat3* = 0.15, s.d. = 0.25). Wilcoxon tests confirmed significant differences among management types, with live-fence individuals showing higher inbreeding than both wild (*f*: *P* = 0.006; *Fhat3*: *P* = 0.003) and cultivated individuals (*Fhat3*: *P* = 0.029) (Table 1).

At the level of individual sampling sites across localities, inbreeding was generally inversely related to genetic diversity (Supplementary Data Table S3). The most inbred site was the live fence from Miahuatlán (*f* = 0.65, s.d. = 0.23), whereas the wild site of San Dionisio Ocotlán was highly outbred (*f* = −0.20, s.d. = 0.007). Some wild sites were exceptions, showing moderate to high inbreeding, such as Miahuatlán (*f* = 0.39, s.d. = 0.24), Zapotitlán Salinas (*f* = 0.23, s.d. = 0.10) and San Gabriel Casablanca (*f* = 0.41, s.d. = 0.36).

### Genetic differentiation and defining management units

The PCA revealed limited differences among individuals along the first two axes, which explained 8.38 and 4.85 % of the total genetic variance, respectively (Supplementary Data Fig. S1). Genetic variation did not segregate strictly by management type; individuals from wild, live-fence and cultivated systems largely overlapped, forming a continuum rather than discrete clusters, with wild sites encompassing the broadest diversity, suggesting weak genetic structuring among management categories rather than distinct clustering. When considering ethnotaxa, axis 1 separated some individuals identified as *Cuishe* and *Madrecuishe* from the rest, whereas axis 2 distinguished the *Cachitún* ethnotaxa from the Tehuacán–Cuicatlán region. Most other ethnotaxa formed a central cluster with substantial genetic overlap (Supplementary Data Fig. S2).

**Fig. 2.**
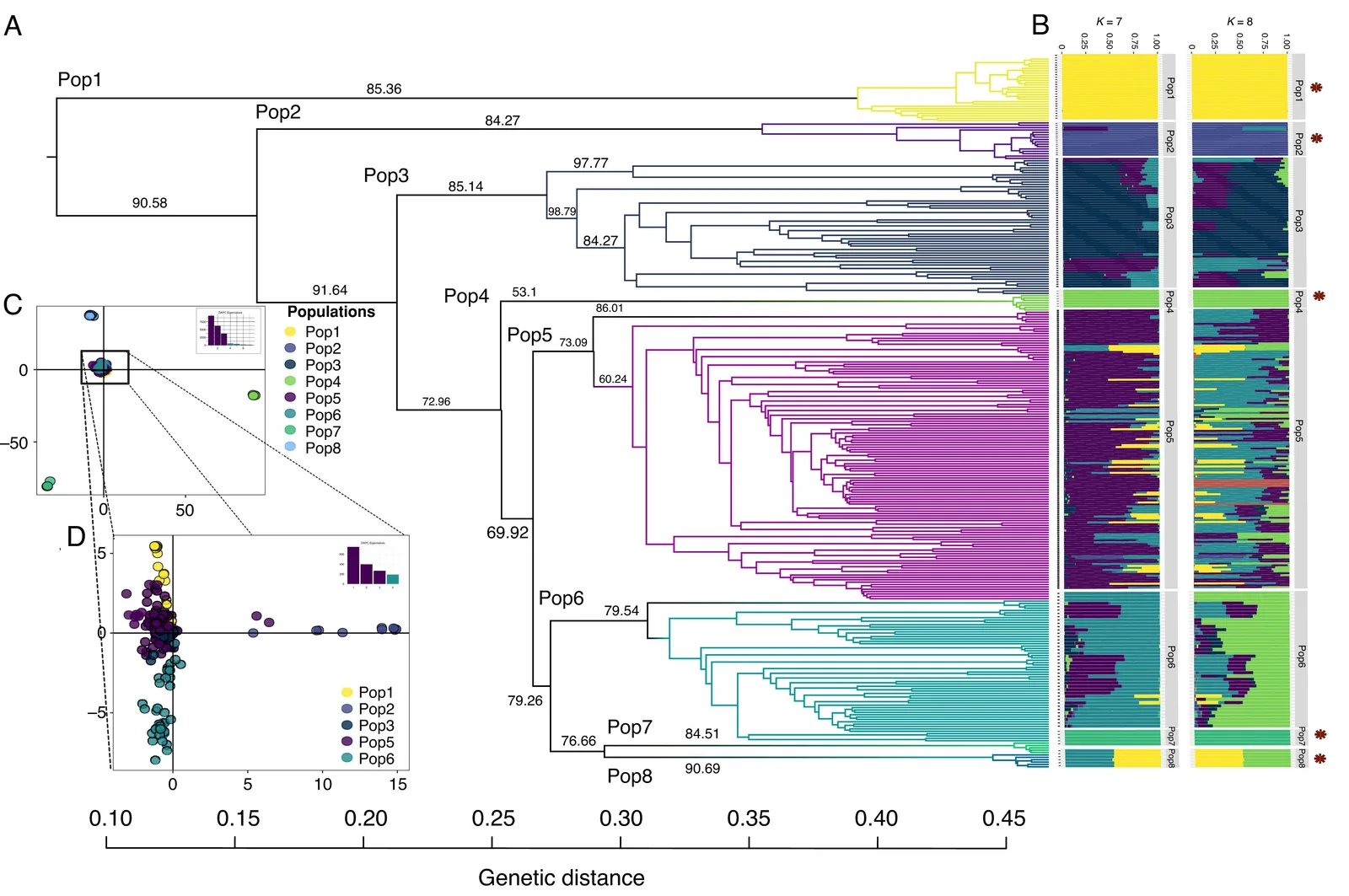
(A) Genetic populations (Pop) of *Agave karwinskii* identified with the UPGMA dendrogram using the poppr package, bootstrap support values are shown at major nodes. (B) Bar plots of individual assignment probabilities for *K* = 7 and *K* = 8 show the proportion of inferred ancestry for each genetic population. (C) Discriminant analysis of principal components (DAPC): the bottom right panel displays the eigenvalues of the discriminant functions. (D) DAPC of the nested genetic populations shown in C. Colours correspond to the clusters identified in the UPGMA dendrogram, and asterisks indicate populations composed of a single ethnotaxa.

The UPGMA dendrogram identified several clusters. We selected the eight most evident, which we designated as genetic populations (Pop; Pop 1–8; Fig. 2A). These clusters broadly reflected both geographical distribution and ethnotaxa, separating northern groups (Tehuacán–Cuicatlán region, Pop 3) from those in the Central Valleys and Sierra Sur districts (Pop 5 and 6). Cultivated ethnotaxa, such as *Madrecuishe*, *Barril Amarillo* and *Barril Verde*, formed distinct clusters (Pop 2, 7 and 8), whereas wild *Cuishe* and *Bicuishe* individuals were distributed across multiple groups. Detailed information on ethnotaxa composition and management context for each genetic population is provided in Table 2.

**Table 2.** Management context, location and ‘ethnotaxa’ for each genetic population of *Agave karwinskii* defined based on genetic clustering analyses (Pop).

Pop	*n*	Management category	Locality	‘Ethnotaxa’
1	27	Wild	Santa María Zoquitlán	*Cuishe*
		Tolerated	Ejutla	*Cuishe*
		*Ex situ* promotion through the transplanting of ramets to create live fences and demarcate plots for crops such as maize or other *Agave* species, such as *A. angustifolia*	Ejutla Santa Catarina Minas San Dionisio Ocotlán Zimatlán	*Tobasiche* *Cuishe*
2	16	*Ex situ* promotion through the transplanting of ramets in varietal gardens	Santa Catarina Minas	*Barril Blanco*
		Cultivated through vegetative propagation	San Luis Amatlán	*Madrecuishe*
3	57	Wild	Zapotitlán Salinas San Gabriel Chilac San José Miahuatlán	*Cachitún*
			San Antonio Nanahuatípam San Juan Bautista Cuicatlán	*Cachitún*
		*Ex situ* promotion through the transplanting of ramets in varietal gardens	Zapotitlán Salinas San Juan Raya	*Cachitún*
		*Ex situ* promotion for established monocultures	Mitla	*Cuishe*
4	7	*In situ* promotion	Coatecas Altas	*Cuishe*
5	141	Wild	Region of Central Valleys	*Cuishe* *Tobasiche*
		Tolerated	Region of Central Valleys	*Cuishe* *Largo*
			Miahuatlán San Luis Amatlán	*Bicuishe* *Largo*
		*Ex situ* promotion for planting as live fences around plots	Region of Central Valleys Miahuatlán San Luis Amatlán	*Cuishe* *Largo* *Tobasiche* *Tripón* *Marteño* *Cirial* *Barril* *Barril de Ejutla*
			Miahuatlán	*Madrecuishe*
		*Ex situ* promotion for planting as live fences around plots and homes and for the establishment of varietal gardens	San Francisco Logueche	*Barril de Ejutla.* *Barril.* *Madrecuishe* *Coyota*
6	41	Tolerated	San Pedro Apóstol (Ocotlán)	*Largo*
		*Ex situ* promotion	Santa Catarina Minas San Dionisio Ocotlán Coatecas Altas San Idelfonso Amatlán Zimatlán	*Cuishe* *Bicuishe* *Barril* *Barril* *Sierra Negra*
		Monocultures established through sexual propagation	Ejutla	*Cuishe*
7	5	*Ex situ* promotion for the establishment of live fences	San Martín Lachila	*Barril Amarillo*
8	6	*Ex situ* promotion for the establishment of live fences	San Martín Lachila	*Barril Verde*

Abbreviation: *n*, number of analysed individuals.

The STRUCTURE analysis corroborated the genetic structure revealed by the UPGMA dendrogram, providing additional resolution for our defined genetic populations (Fig. 2B; Supplementary Data Fig. S3). Although Pop 3, 5 and 6 did exhibit shared alleles, unique genetic components were particularly evident in several populations: *Cuishe* from the Central Valleys (Pop 1), *Madrecuishe* and *Barril Blanco* from the Sierra Sur (Pop 2), *Cuishe* from Coatecas Altas (Pop 4), *Barril Amarillo* from Ejutla (Pop 7), and the genetically mixed *Barril Verde* from Ejutla (Pop 8) (asterisks in Fig. 2B).

The first two discriminant axes of the DAPC explained 46 and 30.1 % of the among-group variation, respectively. Along these axes, four major genetic groupings became apparent: a large central cluster including most populations, whereas Pop 4, 7 and 8 appeared distinct and positioned in peripheral regions of the discriminant space (Fig. 2C). The nested DAPC revealed additional structure within the central genetic grouping identified in the global analysis (Pop 1–3, 5 and 6). With the exception of Pop 3 and 5, which showed marked overlap, the remaining populations, although sharing substantial genetic variation, remain partly differentiated along the first discriminant axes (Fig. 2D).

The genetic differentiation was moderate when individuals were classified by management regimes (*F*_ST_ = 0.08), increased at the level of sites across localities (*F*_ST_ = 0.14) and was highest when using the UPGMA dendrogram-defined population clusters (*F*_ST_ = 0.19). These results highlight a clear division: Pop 1, 2, 4, 7 and 8 were highly differentiated from one another (mean pairwise *F*_ST_ = 0.44), whereas Pop 3, 5 and 6 formed a group with low differentiation (mean pairwise *F*_ST_ = 0.05) (Supplementary Data Fig. S4).

At the individual sampling sites across the locality level, patterns of differentiation varied, ranging from no detectable genetic structure (*F*_ST_ = 0.00), as seen between the wild Miahuatlán and live-fenced Santa Catarina Minas sites, and between some geographically close wild sites (e.g. San Luis Amatlán and San Francisco Logueche), to strong genetic differentiation among several wild sites from the Tehuacán Valley (e.g. Acatepec and San Pedro Tetitlán) (*F*_ST_ = 0.25 to *F*_ST_ = 0.53, respectively). Likewise, the sites of Zapotitlán Salinas in Puebla, regardless of management, were also highly differentiated from the Oaxaca groups, although they exhibited moderate differentiation among themselves (*F*_ST_ = 0.07–0.10). Even within Oaxaca, some wild sites, such as San Dionisio Ocotlán and Zimatlán, displayed strong isolation, with *F*_ST_ values reaching ≤0.49 when compared with other sites (Supplementary Data Fig. S5).

The fineRADstructure heatmap summarizes patterns of pairwise co-ancestry among individuals, reflecting relative levels of recent shared ancestry across populations. Higher within-population co-ancestry was observed in Pop 1, 2, 4, 7 and 8, indicating elevated levels of genetic relatedness among individuals within these groups. Pop 3 showed intermediate levels of within-population co-ancestry relative to the rest of the dataset. In contrast, Pop 5 and 6 exhibited similar co-ancestry patterns and extensive sharing among individuals from both populations, consistent with a high degree of genetic connectivity between them rather than clear differentiation (Supplementary Data Fig. S6). Overall, co-ancestry coefficients were generally higher within populations than among them, and individuals from cultivated and live-fence contexts exhibited more diffuse co-ancestry patterns compared with wild populations.

Analyses restricted to wild individuals, which were associated primarily with genetic Pop 3 (Tehuacán–Cuicatlán region) and Pop 1, 5 and 6 (Central Valleys–Sierra Sur regions), revealed a spatial pattern, with samples arranged along axis 1 in the PCA (12.07 % of variance) according to their geographical origin (Supplementary Data Fig. S7A). Genetic differentiation among these three regions was moderate (*F*_ST_ = 0.10), and co-ancestry patterns from fineRADstructure distinguished individuals from Tehuacán–Cuicatlán (Pop 3) from those in the Central Valleys–Sierra Sur of Oaxaca (Pop 1, 5–6), although overall co-ancestry between regions was low. In contrast, Pop 5 and 6 showed strong internal co-ancestry and formed a closely related group (Supplementary Data Fig. S7B).

Because the inclusion of cultivated and managed individuals did not markedly affect the isolation-by-distance results, we focused on analyses restricted to wild individuals. This subset revealed a clearer spatial genetic structure and a stronger isolation-by-distance pattern (*r*^2^ = 0.18, *P* = 0.001 for genetic distance; *r*^2^ = 0.25, *P* = 0.05 for *F*_ST_), consistent with the geographical separation between the Central Valleys–Sur Sierra and Tehuacán–Cuicatlán regions (Supplementary Data Fig. S7C). No significant association with elevation was detected (*P* > 0.05).

When we analysed the genetic diversity of each defined genetic population (Table 3), two contrasting genetic profiles emerged. The first group (Pop 1, 2, 4, 7 and 8) maintained high heterozygosity (*H*_o_ ≥ 0.25; MLH ≈ 0.25–0.27) and negative inbreeding coefficients (*F*_IS_ ≈ −0.19 to −0.23), indicating an excess of heterozygotes despite lower nucleotide diversity (π ≈ 0.15). The second group included Pop 5 and 6 exhibited higher nucleotide diversity (π = 0.23–0.25) lower heterozygosity (*H*_o_ ≈ 0.22; MLH ≈ 0.21) and mild inbreeding (*F*_IS_ = 0.07–0.09; *f* ≈ 0.14–0.18). Population 3 represented a unique case, combining the lowest individual heterozygosity (*H*_o_ = 0.21) with the highest inbreeding level (*f* ≈ 0.27), yet retaining high total diversity (*H*_s_ and π ≈ 0.22). Private alleles were detected only in two populations: Pop 3 (four alleles) and 5 (14 alleles).

**Table 3.** Genetic diversity parameters for the genetic groups (Pop; see main text) of *Agave karwinskii*.

Pop	*n*	*H*_o_	*H*_s_	MLH	sMLH	*F*_IS_	*f*	*Fhat3*	π
1	27	0.28 (0.41)	0.15 (0.22)	0.25 (0.08)	1.10 (0.35)	−0.23 (0.13)	−0.01 (0.32)	0.01 (0.27)	0.15 (0.17)
2	16	0.26 (0.39)	0.15 (0.21)	0.25 (0.04)	1.11 (0.17)	−0.20 (0.12)	−0.01 (0.16)	−0.01 (0.15)	0.15 (0.16)
3	57	0.21 (0.17)	0.22 (0.17)	0.18 (0.06)	0.79 (0.29)	0.08 (0.07)	0.27 (0.26)	0.26 (0.28)	0.21 (0.12)
4	7	0.26 (0.43)	0.13 (0.22)	0.26 (0.00)	0.14 (0.00)	−0.22 (0.13)	−0.04 (0.00)	−0.03 (0.0)	0.14 (0.17)
5	141	0.22 (0.13)	0.25 (0.13)	0.21 (0.07)	0.92 (0.31)	0.09 (0.02)	0.14 (0.28)	0.16 (0.24)	0.23 (0.10)
6	41	0.21 (0.15)	0.23 (0.15)	0.20 (0.04)	0.88 (0.20)	0.07 (0.05)	0.18 (0.18)	0.15 (0.17)	0.21 (0.12)
7	5	0.25 (0.43)	0.12 (0.21)	0.25 (0.00)	1.0 (0.00)	−0.19 (0.11)	0.01 (0.00)	0.02 (0.00)	0.14 (0.10)
8	6	0.27 (0.43)	0.13 (0.22)	0.27 (0.00)	1.16 (0.02)	−0.22 (0.13)	−0.06 (0.02)	−0.04 (0.00)	0.15 (0.10)

The s.d. is shown in parentheses. Abbreviations: *f*, inbreeding coefficient; *Fhat3* index; *F*_IS_, Wright’s inbreeding coefficient; *H*_o_, observed heterozygosity; *H*_s_, expected heterozygosity; *H*_T_, total heterozygosity; MLH, multilocus heterozygosity; *n*, number of individuals per dataset; sMLH, standardized multilocus heterozygosity; π, standardized nucleotide diversity.

### Local adaptation analyses

**Fig. 3.**
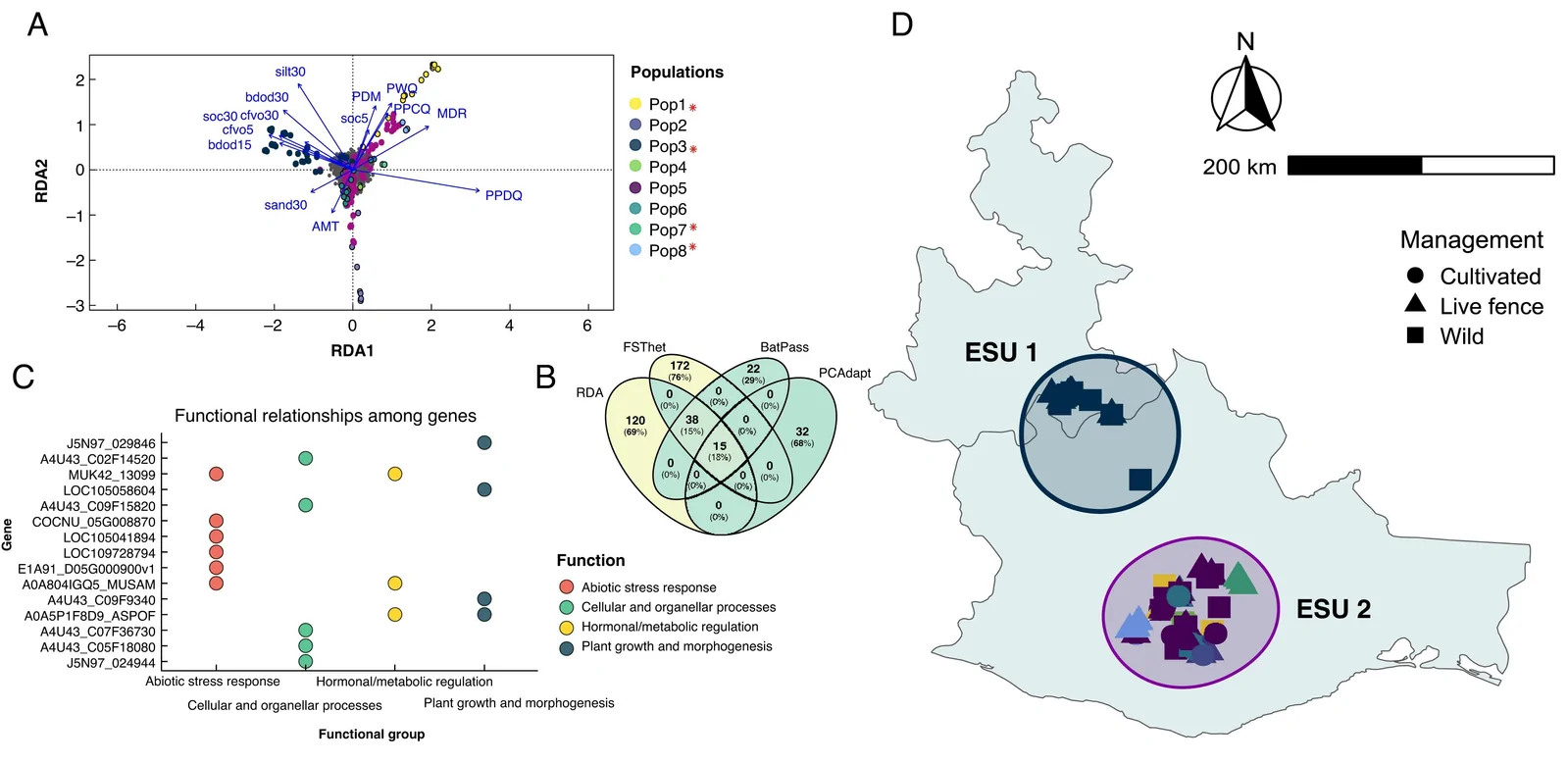
(A) Triplot of the first two redundancy analysis axes, showing SNPs potentially associated with different environmental variables. Grey points at the centre of the diagram represent SNPs. Larger points correspond to individuals, which are coloured according to the genetic groups identified in the UPGMA analysis (Pop). Red asterisks indicate populations composed of a single ethnotaxon (shown in Fig. 2). Blue vectors represent the 14 environmental predictors used in the analysis (Supplementary Data Table S2). (B) Venn diagram showing the overlap of candidate SNPs identified by different detection methods, with 15 consensus SNPs retained for annotation. (C) Annotation of these 15 candidate loci, referenced to an *Agave tequilana* transcriptome, revealed several functional associations. (D) Geographical distribution of genetic groups across *Agave karwinskii* distribution, highlighting two proposed evolutionarily significant units (ESU 1 and ESU 2) defined by the integration of neutral and adaptive loci.

The RDA constrained by 14 environmental predictors explained a significant fraction of the genetic variance (15 %; adjusted *R*^2^ = 0.10), and the overall model was significant based on permutation tests (*P* = 0.001). A total of 173 SNPs were identified as candidate loci potentially associated with environmental variation, most associated with climatic (82) and edaphic (55) variables (precipitation, soil organic carbon and silt content). Pop 3 was influenced mainly by edaphic factors (Leptosol and Regosol soils), whereas Oaxaca populations (Pop 1, 7, 8 and part of 5) were associated with precipitation. Conversely, Pop 6 and the remaining individuals of Pop 5 were correlated with mean annual temperature and soil texture (e.g. sand content) (Fig. 3A).

The different methods used to detect candidate loci revealed varying numbers of outlier SNPs. RDA identified 173 SNPs, and the FSThet approach found 225 SNPs, whereas BayPass detected 75, and the PCAdapt method identified the fewest number of outliers, 47 SNPs (Fig. 3B).

Based on methodological convergence, 15 robust SNPs (identified by the four detection approaches) were annotated using the *A. tequilana* transcriptome (Fig. 3B). All loci were matched successfully in UniProt to genes involved in defence responses, adaptation to environmental stressors (e.g. drought, salinity, temperature extremes, ultraviolet radiation, oxidative stress), growth and metabolic regulation (Fig. 3C; Supplementary Data Table S4).

### Estimation of the effective population size

Estimates of *N*_e_ varied considerably across genetic populations and between methods. NeEstimator produced markedly lower *N*_e_ values in most populations. For instance, estimates were as low as *N*_e_ = 0.5 in Pop 2, with higher values in more admixed populations, such as Pop 5 (*N*_e_ = 17.5) and 6 (*N*_e_ = 13.4). In contrast, the SA method (Colony2) yielded higher *N*_e_ estimates, such as *N*_e_ = 139 in Pop 5 and *N*_e_ = 89 in Pop 6. Although the Wilcoxon signed-rank test revealed no statistically significant difference between methods (*P* = 0.06), results suggest a consistent trend towards higher values of *N*_e_ produced by Colony2 (Supplementary Data Table S5).

When the genetic components were subdivided by management type, a two-way ANOVA revealed that both management type (*P* = 0.001) and estimation method (*P* < 0.001) significantly influenced *N*_e_ values, whereas their interaction was not significant (*P* = 0.205) (Supplementary Data Table S6). The highest *N*_e_ estimates were obtained in *ex situ*-promoted populations (≤156 with Colony2), whereas vegetatively propagated populations showed the lowest values, approaching zero (e.g. 0.6 in Pop 2 with NeEstimator). Considering the approximate census size (*N*_c_) derived from field observations, the *N*_e_/*N*_c_ ratio ranged from 0.29 in the *ex situ* group (Pop 3) to 0.00005 in the vegetatively propagated Pop 2 (Table 4; Supplementary Data Table S7).

**Table 4.** Contemporary *N*_e_ estimates for *Agave karwinskii* genetic groups (Pop; see main text) subdivided by management type.

Pop	Type of management	*n*	*N*_c_ (Obs)	*N*_e_ in NeEstimator (95 % CI)	*N*_e_/*N*_c_	*N*_e_ in Colony2 (95 % CI)	*N*_e_/*N*_c_
1	*Ex situ* promotion	14	130	3 (0.3–infinite)	0.02 (0.002–NA)	3 (2–infinite)	0.02 (0.01–NA)
1	Wild	13	150	3 (1.2–112.9)	0.02 (0.001–0.75)	4 (2–17)	0.02 (0.01–0.1)
2	Cultivated by vegetative propagation	16	12 000	0.6 (0.4–0.7)	0.00005 (0.00003–0.00006)	3 (2–infinite)	0.0002 (0.0001–NA)
3	*Ex situ* promotion	19	75	1.3 (0.6–5.3)	0.01 (0.008–0.07)	22 (10–45)	0.29 (0.13–0.6)
3	Wild	38	900	3.5 (2.4–11.2)	0.003 (0.002–0.01)	23 (13–45)	0.03 (0.01–0.05)
5	Cultivated by vegetative propagation	20	700	8.3 (2.9–137.9)	0.01 (0.004–0.20)	26 (14–46)	0.04 (0.02–0.06)
5	*Ex situ* promotion	60	3500	20.9 (3.8–90.3)	0.005 (0.001–0.02)	156 (107–224)	0.04 (0.03–0.06)
5	Tolerated	25	350	23.2 (8.1–32.2)	0.06 (0.02–0.09)	78 (47–150)	0.22 (0.13–0.42)
5	Wild	25	600	10.6 (9.2–infinite)	0.01 (0.01–NA)	48 (27–85)	0.08 (0.04–0.14)
6	*Ex situ* promotion	20	200	15.3 (7.2–infinite)	0.08 (0.03–NA)	42.5 (23–84)	0.21 (0.11–0.42)
6	Cultivated by vegetative propagation	9	750	1.9 (0.6–infinite)	0.002 (0.0008–NA)	18.5 (7–52)	0.02 (0.01–0.07)

Abbreviations: CI, confidence interval; *n*, number of samples analysed; *N*_c_ Obs, approximation of the census size based on direct field observation; NA, not available.

## DISCUSSION

Using the genomic data generated, we were able to characterize the genetic diversity, population structure and inbreeding and to delimitate conservation units for *A. karwinskii*. In addition, we estimated the effective population size across sampling sites in different localities with different management regimes and expected levels of clonality, which is required to report on the GBF headline genetic indicator (proportion of populations within the species with an *N*_e_ > 500) (Mastretta-Yanes *et al.*, 2024*a*).

### Genetic diversity in *A. karwinskii*

Similar to other mezcal-producing agave species recently introduced into intensive management, such as *Agave potatorum* Zucc., *A. marmorata* Roezl. and *A. angustifolia* var. *pacifica*, *A. karwinskii* exhibits a comparable level of overall genetic diversity (*H*_s_ = 0.25) (Klimova *et al.*, 2022, 2023; Ruiz Mondragón *et al.*, 2023, 2024). This value is also similar to those reported for the wild *Agave aurea* complex (Klimova *et al.*, 2024) and *Yucca* spp. from Baja California (Arteaga *et al.*, 2020). Moreover, it resembles levels found in other partly clonal systems, such as the orchid *Cypripedium calceolus* (Gargiulo *et al.*, 2023), but exceeds those observed in *A. tequilana* Weber (*H*_s_ = 0.12) and some *A. angustifolia* varieties (*H*_s_ = 0.13), both strongly shaped by long-term clonal propagation and intensive management (Cabrera-Toledo *et al.*, 2022; Ruiz Mondragón *et al.*, 2022).

This pattern is likely to reflect the recent onset of intensive management, whereby the studied managed populations still retain high levels of genetic variation. Additionally, clonal propagation can preserve heterozygous genotypes, and given that wild agaves are often partly clonal, similar diversity levels do not necessarily imply the absence of genetic erosion. Moreover, factors other than management, such as somatic mutation (Klimova *et al.*, 2023) and a possible hybrid origin (Montiel-Castelán *et al.*, 2025), can also contribute to elevated genetic diversity in wild and cultivated agaves, highlighting that several evolutionary processes might underlie observed patterns.

Wild sites of *A. karwinskii* harbour the highest genetic variability, and the overall heterozygosity and nucleotide diversity remain relatively high and homogeneous across cultivated and live-fence sites. This pattern is consistent with a recent shared origin between wild and managed groups, the recent nursery-grown seedlings and the frequent exchange of plant material among producers, as documented through field observations and information provided by local communities during sampling and supported by previous studies (Vázquez-Pérez, 2015). Additionally, long-distance gene flow mediated by pollinators might also contribute to the genetic connectivity observed among nearby populations (Ruiz Mondragón *et al.*, 2022), as discussed below.

Nevertheless, the vegetative propagation of *A. karwinskii* has led to genetic substructuring within management categories. At this level, inbreeding coefficients (*F*_IS_) were similar between wild and cultivated sites. However, individual-based metrics (*f* and *Fhat3*) revealed substantially higher inbreeding in cultivated sites (approximately twice that of wild groups) and even stronger in live-fence sites. This discrepancy indicates that *F*_IS_ underestimates inbreeding in clonally propagated crops. *F*_IS_ equally weights all alleles, whereas *f* and *Fhat3* are based on correlations and covariances among genetic effects, placing greater weight on homozygosity at rare alleles (Alemu *et al.*, 2021).

Genetic results from live-fence sites are consistent with their predominant clonal propagation from a few founder genotypes. Although live fences have long been integral to Mesoamerican agriculture, serving historically as boundary markers (100–700 Ce) and later as protection against livestock (Zizumbo-Villarreal *et al.*, 2013), they now represent a management system where clonal reproduction severely limits genetic renewal. Despite the overall stability of genetic diversity in wild sites, high inbreeding was detected in localities such as Zapotitlán Salinas, Cuicatlán and Miahuatlán. In the Tehuacán–Cuicatlán Valley, habitat fragmentation and overharvesting have resulted in isolated populations (Flores, 2022), whereas in Miahuatlán, plantation expansion and urban growth have increased genetic isolation further. These factors, together with clonal reproduction and the naturally patchy distribution of *A. karwinskii*, are likely to underlie the elevated inbreeding observed in these regions.

### Genetic differentiation in *A. karwinskii*

Unlike most agave species, which exhibit low genetic structure (Eguiarte *et al.*, 2013), *A. karwinskii* shows clear genetic differentiation, apparently shaped by geographical, ecological and anthropogenic factors. Across all analyses, clustering patterns consistently revealed multiple hierarchical levels of genetic structure. Although scenarios with seven to eight clusters best captured the differentiation among ethnobotanical groups and management units, alternative interpretations based on Δ*K* values and pairwise differentiation suggested broader genetic groupings, particularly among Pop 3, 5 and 6, where extensive admixture indicates ongoing gene flow.

This structure largely corresponds to the species distribution across two major ecological regions, the Tehuacán–Cuicatlán Valley and the Central Valleys–Sierra Sur of Oaxaca, which are separated by only ∼75 km, yet divided by a clear genetic discontinuity. Neutral SNPs reveal ongoing divergence driven by both gradual isolation and reduced connectivity. Wild sites in Acatepec, San Pedro Tetitlán (Tehuacán–Cuicatlán), San Dionisio Ocotlán and Zimatlán (Oaxaca) exhibit high differentiation (*F*_ST_ = 0.25–0.53), indicating long-term reproductive isolation.

The fine-scale isolation is embedded within broader patterns of connectivity. At the cluster level, divergent Tehuacán populations still group within larger genetic units that show low differentiation from central and sur Oaxaca clusters (*F*_ST_ = 0.06–0.07). This suggests that shared ancestry has been maintained by historical and ongoing gene flow, consistent with bat-mediated pollination, typically associated with high gene flow and low structure in agaves (Eguiarte *et al.*, 2013; Ruiz Mondragón *et al.*, 2022). However, emerging differentiation is evident, reinforced by ecological barriers, such as flowering asynchrony (Vázquez-Pérez, 2015), and, crucially, by human management practices.

The relationship between humans and *A. karwinskii* shapes its genetic landscape along management gradients that both intensify divergence and, paradoxically, homogenize it. Clonal propagation and restricted sexual reproduction drive strong differentiation in managed sites, leading to reduced nucleotide diversity relative to heterozygosity (Klimova *et al.*, 2023). However, excess heterozygosity might also reflect the selective propagation of vigorous or heterozygous individuals, as observed in the most divergent ethnotaxa (e.g. *Cuishe* from Coatecas Altas, *Madrecuishe* and some *Barriles*), which differ in traits such as stem presence, spine morphology, leaf dentition and coloration (Vázquez-Pérez *et al.*, 2015). Similar patterns of genetic differentiation have been reported in *A. angustifolia* landraces from Jalisco, which have been reproduced vegetatively for generations under traditional management (Cabrera-Toledo *et al.*, 2022), highlighting the parallel evolutionary outcomes of prolonged clonal cultivation in agaves.

Regional management practices modulate these patterns further. In Tehuacán–Cuicatlán Valley of Puebla, the absence of intensive monocultures and reliance on nearby wild stocks maintain regional genetic identity, with low differentiation among sites (*F*_ST_ = 0.07) regardless of management. Conversely, in Oaxaca, semi-intensive practices, such as live fences and long-term clonal propagation, have produced distinct genetic clusters, such as *Cuishe* from Coatecas Altas and *Barril* from San Martín Lachilá, reflecting early domestication driven by selection for robust, tall individuals (Rivera, 1983). As in other vegetatively propagated crops, such processes might fix plastic traits through genetic assimilation and sustained human selection (Denham *et al.*, 2020). *Madrecuishe* from Miahuatlán suggests this trajectory: maintained for generations by mezcal producers (Urrutia-Cruz, 1986), it shows pronounced genetic divergence (*F*_ST_ = 0.21), consistent with previous morphological and genetic findings (Vázquez-Pérez, 2015) and comparable to patterns in *A. angustifolia* ‘Espadín’ (Klimova *et al.*, 2023).

At the same time, human-mediated movement of plant material homogenizes genetic differentiation, explaining clusters that do not match geographical origin; for instance, a *Barril Blanco* from Ocotlán clustering with *Madrecuishe* from Miahuatlán. In contrast, wild sites display genetic patterns structured primarily by geography, with clear isolation by distance and spatial coherence in the absence of human-mediated dispersal. This contrast underscores how management and exchange promote genetic mixing, whereas natural sites retain fine-scale differentiation.

The high allele frequency and nucleotide diversity observed in the large Oaxaca populations (Pop 5 and 6) reflect a mosaic of ethnotaxa and management intensities, making them key reservoirs for conservation and sustainable use. This supports the broader view that traditional management can act as a form of *in situ* conservation, maintaining and enhancing genetic diversity (Casas *et al.*, 1999; Vázquez-Pérez, 2015; Mastretta-Yanes *et al.*, 2024*b*). In contrast, Pop 3, although mostly wild, exhibits genetic subdivision and inbreeding, which are patterns that could become critical under climate change, as seen in other agaves, such as *A. angustifolia* and *Agave victoriae-reginae* (Aguirre-Dugua and Eguiarte, 2013).

### Estimation of the contemporary effective population size

Given that most agaves are semelparous, differences in *N*_e_ depend strongly on variance in reproductive success. Anthropogenic management introduces additional complexity: wild extraction reduces census size (*N*_c_), whereas live fences and monocultures suppress recombination, create fixed heterozygosity and decouple *N*_c_ from *N*_e_ (Gargiulo *et al.*, 2023; Klimova *et al.*, 2023).

In *A. karwinskii*, this disconnection is evident. Despite large *N*_c_ in some populations, estimates of *N*_e_ were consistently low; well below the GBF threshold of *N*_e_ > 500 (indicator A.4, Target 4; Convention on Biological Diversity (CBD; www.cbd.int)), aligning instead with values typical of partly clonal plants (*N*_e_ < 50) (Gargiulo *et al.*, 2023), where the risk of inbreeding depression and elevated extinction risks are expected in the long term (Frankham *et al.*, 2014). For instance, the biggest wild population of *A. karwinskii*, located in the Tehuacán–Cuicatlán Reserve (Valiente-Banuet, 2023), had the higher *N*_c_ but lower *N*_e_ than those in Oaxaca, consistent with the effects of clonal aggregation and geitonogamy (i.e. mating between ramets of the same genet), leading to reduced *N*_e_ and increased inbreeding (Gargiulo *et al.*, 2023).

Extremely low *N*_e_/*N*_c_ ratios (0.00004–0.29) highlight severe genetic erosion, because *N*_c_ counts both ramets and genets, whereas *N*_e_ reflects only unique genets. This explains why demographically abundant populations can be genetically vulnerable. Conversely, *ex situ* promotion and tolerated management yielded higher values of *N*_e_ (≤156 and ≤78, respectively). These findings remark the potential of *ex situ* conservation practices to act as reservoirs of genetic diversity and, if managed appropriately, to contribute to increasing *N*_e_ in declining populations. However, such strategies should be designed carefully to avoid unintended genetic homogenization (as monocultures) or maladaptation upon reintroduction.

The proportion of maintained populations (PM indicator) within species, which quantifies population retention as a proxy for preserving interpopulation genetic diversity (Mastretta-Yanes *et al.*, 2024*a*), indicated complete population retention in *A. karwinskii* (PM = 1), because all historically reported populations remain extant according to our field observation (Rivera, 1983; Vázquez-Pérez *et al.*, 2015). Yet this estimate masks recent demographic contractions and overharvesting, with most values of *N*_e_ falling below thresholds needed to preserve evolutionary potential. Thus, presence-based metrics alone overestimate resilience in partly clonal species. Conservation assessments must therefore integrate estimates of *N*_e_ (particularly relative differences across management contexts) with demographic data to capture the true genetic risks facing *A. karwinskii*.

In light of these findings, we emphasize that conservation assessments for partly clonal species under management should rely not only on absolute *N*_e_ thresholds, but also on relative comparisons across management types and local demographic contexts. This is particularly important when using transcriptome-derived SNPs, which are located exclusively in coding regions of the genome and can bias LD-based estimates of *N*_e_ downwards owing to elevated LD and reduced genetic representation (Luikart *et al.*, 2003).

Despite these potential biases, transcriptome-based SNPs offer high genotyping accuracy and low error rates, which are advantageous for pedigree-based methods, such as those implemented in Colony2 (Harney *et al.*, 2018). Importantly, the combined use of two independent methods, LD and SA, in addition to our emphasis on relative comparisons across genetic components and management regimes, mitigates many of the biases inherent to transcriptomic datasets.

### Conservation genetics of *A. karwinskii*

One of the more innovative elements of the Convention on Biological Diversity (CBD; www.cbd.int) Kunming–Montreal Global Biodiversity Framework (GBF) is the explicit inclusion of genetic diversity (Hoban *et al*., 2020; Mastretta-Yanes *et al.*, 2024*a*). Therefore, monitoring and reporting the genetic health of wild and managed populations of species is essential for providing indicators that inform international (i.e. IUCN Red List; https://www.iucnredlist.org/) and national (i.e. the Mexican SEMARNAT NOM-059; https://www.gob.mx/semarnat) conservation policies. However, the conservation of genetic diversity in plant systems with high cultural and economic value is challenging, because human-guided evolutionary forces operate simultaneously with natural ones, and the balance between these forces shapes the resulting evolutionary processes (Casas *et al.*, 2024).

Given the high levels of genetic variation (*H*_s_ = 0.25) and population differentiation (*F*_ST_ = 0.19), *A. karwinskii* falls within Quadrant IV of the conservation model proposed by Eguiarte *et al.* (2013). In this category, preservation of both the largest possible number of individuals and of populations is essential to maintain overall genetic diversity.

From the previously mentioned information, and given the dual scenario, in which ecological barriers shape differentiation among wild populations and management intensifies divergence in cultivated contexts, we propose that the identified genetic populations be recognized as independent MUs. This proposal is supported by the observed levels of genetic differentiation among populations, the patterns recovered by clustering analyses, and the correspondence between several genetic populations and locally recognized ‘ethnotaxa’ associated with distinct morphological selection practices across regions. However, the low genetic differentiation (*F*_ST_ = 0.03), together with the similarity in ethnotaxa composition and management practices between Pop 5 and 6, suggests that these groups form a genetically continuous unit within Oaxaca and are therefore treated as a single MU for conservation purposes.

This proposal, supported by genetic differentiation, is reinforced by signals of local adaptation. Candidate loci associated with environmental variables suggest that adaptation in *A. karwinskii* is driven primarily by edaphic factors in the Tehuacán–Cuicatlán region and by precipitation–temperature gradients in Central Valleys–Sur Sierra in Oaxaca. In Tehuacán, nutrient-poor but well-drained soils are likely to promote adaptation by enhancing water flow and reducing competition, consistent with previous studies (Valiente-Banuet *et al.*, 2000).

Local adaptation maintains phenotypic diversity and fosters genotypic differentiation along environmental gradients (Merilä and Crnokrak, 2001); historically, these processes have shaped the evolution of agaves (Rivera, 1983). Accordingly, the combined evidence from geographical structure, co-ancestry analyses, isolation-by-distance patterns and signatures of genetic–environmental association support the recognition of two evolutionarily significant units: populations from the Tehuacán–Cuicatlán region (ESU 1) and those from Oaxaca (ESU 2) (Fig. 3D). Within ESU 2, the proposed MUs comprise a mosaic of wild and managed populations, forming a complex conservation landscape where traditional management and natural selection interact.

We identified 15 consensus loci as strong candidates for local adaptation, with predicted functions related to the stress response and structural development (see Supplementary Data Table S4; Supplementary References). Similar functional categories have been reported in genomic studies of monocot crops and perennial species (Xu et al., 2012; Jiao *et al.*, 2020: listed in the Supplementary References), supporting the biological plausibility of the candidate loci identified here. These loci provide a valuable genetic framework for conservation and for assessing the adaptive potential of the species under climate change. Nonetheless, given that our analysis is based on transcriptomic references, further whole-genome studies are needed to confirm their roles and clarify their association with the proposed MUs and evolutionarily significant units.

### Conclusion

The case of *A. karwinskii* highlights the value of integrating genomic data and evolutionary analyses into traditional management to ensure the long-term sustainability of mezcal-producing landscapes. Conservation and restoration strategies should operate at the level of evolutionarily significant units, maintaining the genetic integrity of Puebla and Oaxaca populations and preventing maladaptive mixing. Reforestation efforts must focus on expanding natural populations to reduce inbreeding and promote seed-based regeneration. Within MUs, the inclusion of sexual reproduction should be encouraged to preserve adaptive potential, while nurseries might serve as reservoirs of genetic diversity rather than substitutes for traditional clonal propagation. The sampling strategy used here proved effective for capturing genetic variability and can guide future conservation planning. Alignment of genetic insights with local practices will strengthen both the biological resilience of *A. karwinskii* and the cultural heritage associated with its use.

## Supplementary Material

mcag132_Supplementary_Data

## Data Availability

The data underpinning our analyses have been deposited in an online repository (http://www.ncbi.nlm.nih.gov/bioproject/1362929).

## SUPPLEMENTARY DATA

Supplementary data are available at *Annals of Botany* online and consist of the following. Table S1: sampling site information. Table S2: environmental layers included in the genotype–environment association analysis using a redundancy analysis. Table S3: genetic diversity and inbreeding estimates across management types. Table S4: functional annotation and characteristics of consensus candidate single nucleotide polymorphisms identified as putatively under selection. Table S5: contemporary effective population size (*N_e_*) estimates for genetic groups. Table S6: results of the two-way ANOVA testing the effects of management type and estimation program on log-transformed contemporary effective population size (*N*_e_) estimates. Table S7: contemporary effective population size (*N_e_*) estimates for genetic groups partitioned by management type. Supplementary references: scientific sources associated with UniProt matches from BLASTx annotation of candidate single nucleotide polymorphism-containing consensus sequences (Supplementary Data Table S4). Figure S1: principal component analysis plot showing genetic variation patterns of 300 *Agave karwinskii* individuals, according to management type. Figure S2: principal component analysis plot showing genetic variation patterns based on locally recognized ethnotaxa and shaped by management type. Figure S3: STRUCTURE results for *K* = 2–6 and Δ*K* analysis from Structure Harvester identifying the most likely number of genetic clusters. Figure S4: pairwise *F*_ST_ matrix among genetic groups identified through UPGMA clustering. Figure S5: pairwise *F*_ST_ matrix among sampled populations of *Agave karwinskii* from Puebla and Oaxaca. Figure S6: fineRADstructure co-ancestry heatmap showing patterns of genomic relatedness among 300 individuals based on shared loci. Figure S7: genetic structure of 120 wild individuals illustrated through principal component analysis, fineRADstructure co-ancestry heatmap, and isolation-by-distance analysis across Puebla and Oaxaca.
