# Supplementary material for: Clonality, management and geography shape genetic structure in a perennial species with restricted distribution
Source: Ann Bot. 2026 May 15;138(4):1223–38. doi: 10.1093/aob/mcag132 (PMC13595273; doi:10.1093/aob/mcag132)
Supplement: Supplementary file 1 [file mcag132_supplementary_data.pdf]

Supplementary Information for

Clonality, management, and geography shape genetic structure in a perennial species with restricted distribution.

Supplementary Figures

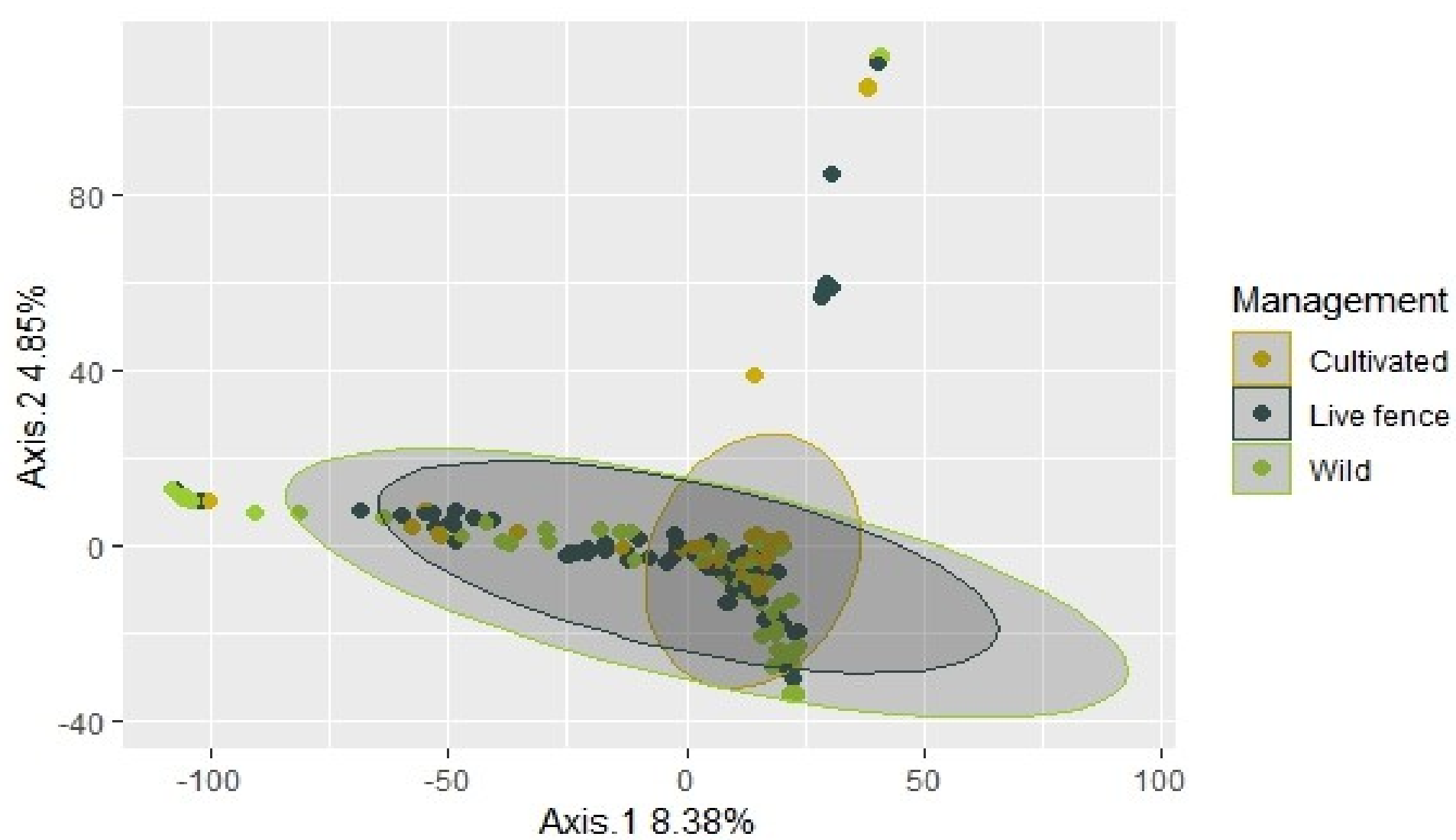

Figure S1. Principal Component Analysis (PCA) of 300 *Agave karwinskii* individuals based on 7, 156 SNPs. Each point represents an individual, colored according to its management type. Shaded ellipses represent the 99% confidence intervals for each group. The percentage of total variance explained by the first two principal components is indicated on the respective axes.

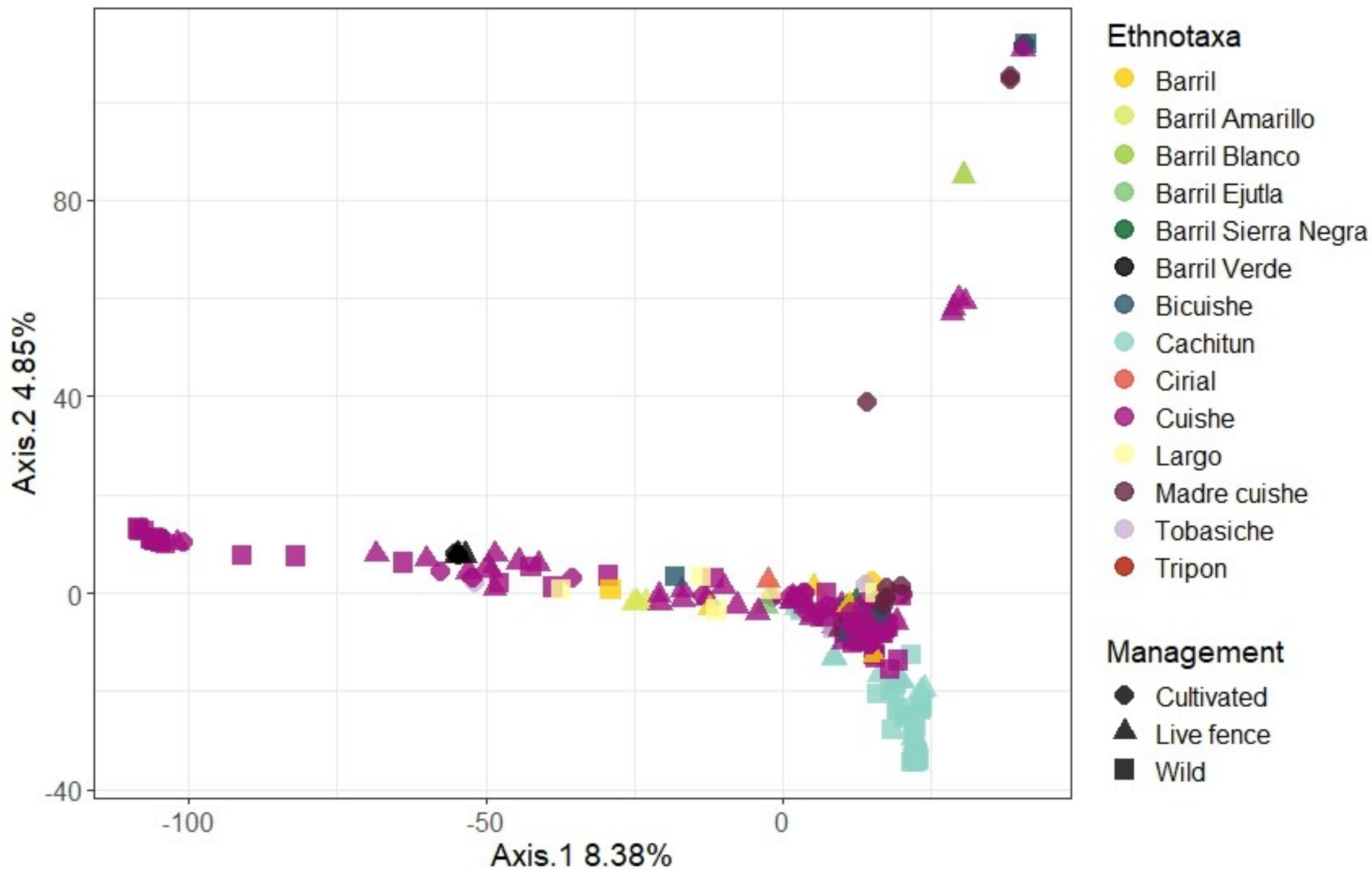

Figure S2. Principal Component Analysis (PCA) of 300 *Agave karwinskii* individuals based on 7, 156 SNPs. Each point represents an individual, colored by its locally recognized ethnotaxa and shaped by its management type. The percentage of total variance explained by the first two principal components is indicated on the respective axes.

A

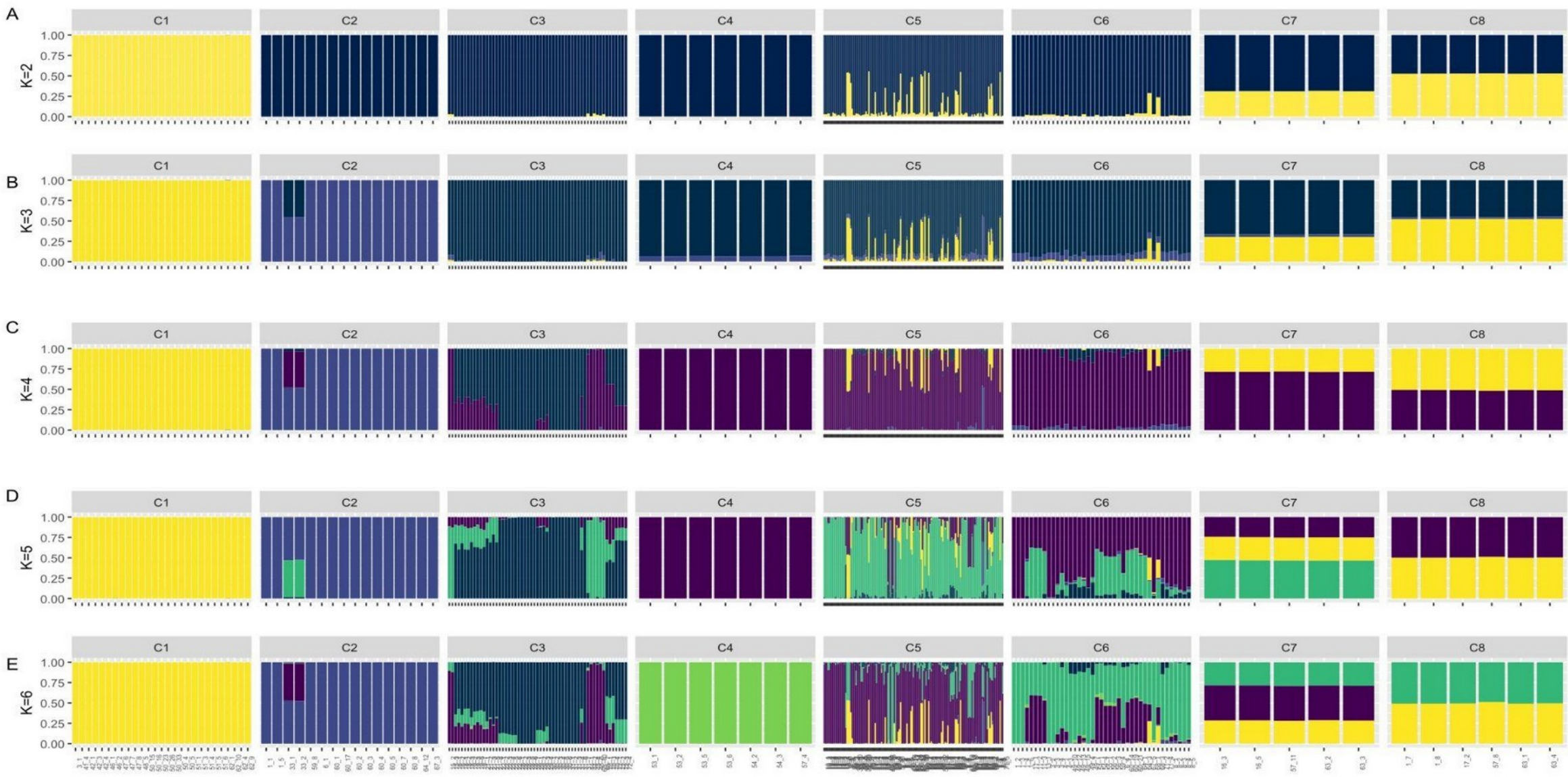

B

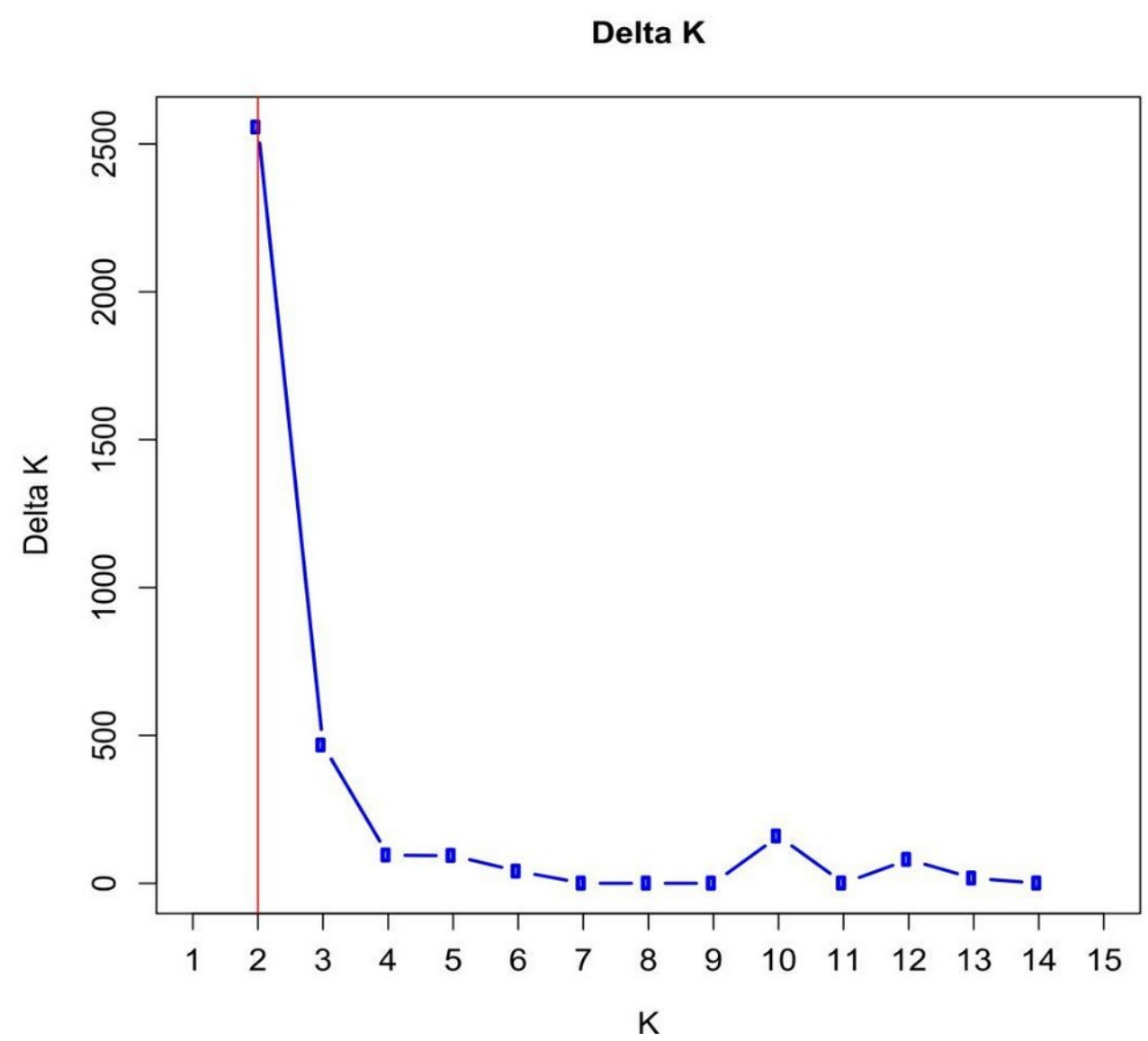

Figure S3. STRUCTURE results for $K = 2$ to $K = 6$ based on 300 individuals of *Agave karwinskii* and 7,156 SNPs (A). Results from Structure Harvester identifying the most likely number of genetic clusters based on ΔK analysis (B).

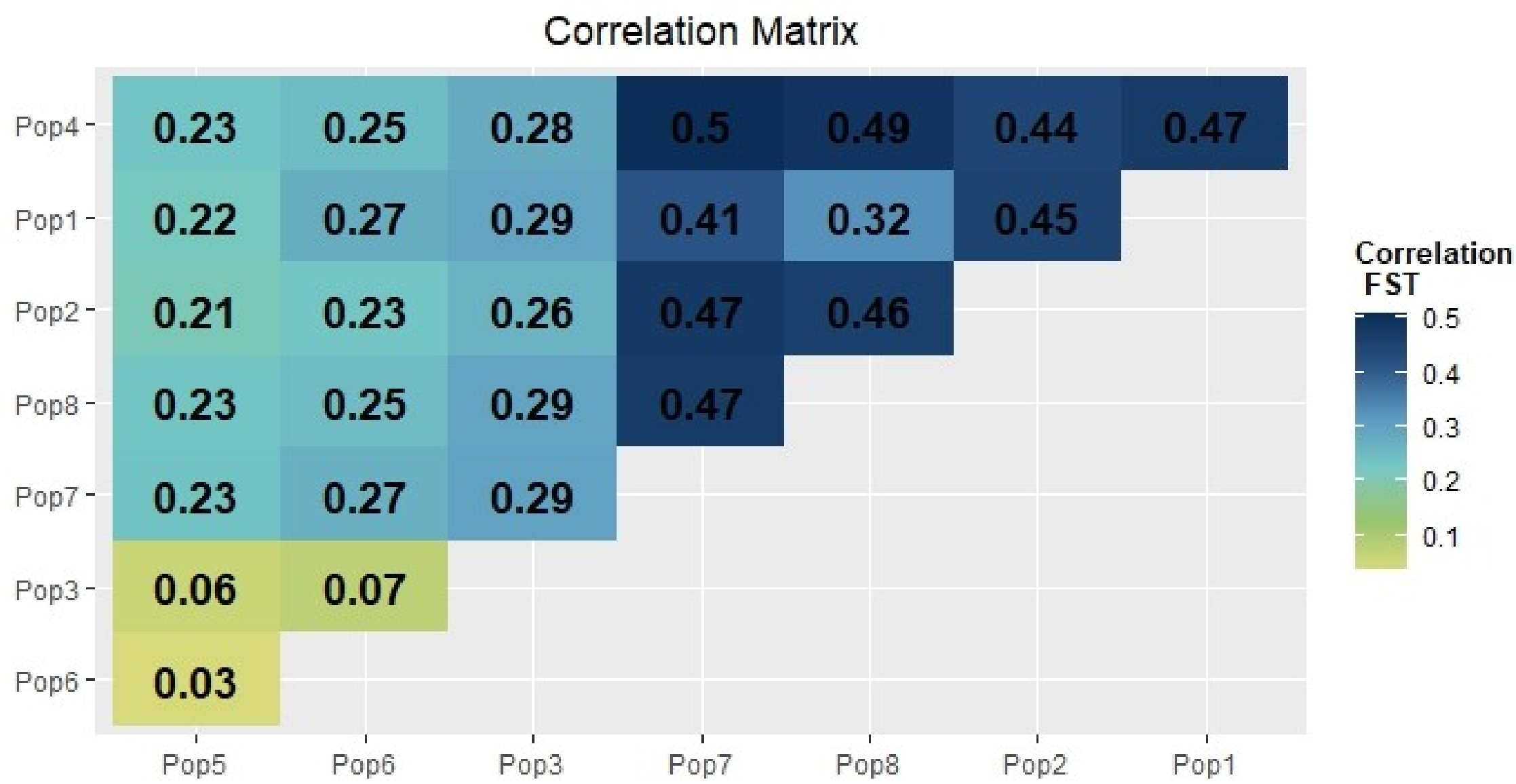

Figure S4. Pairwise F_{ST} matrix among genetic groups identified using UPGMA clustering.

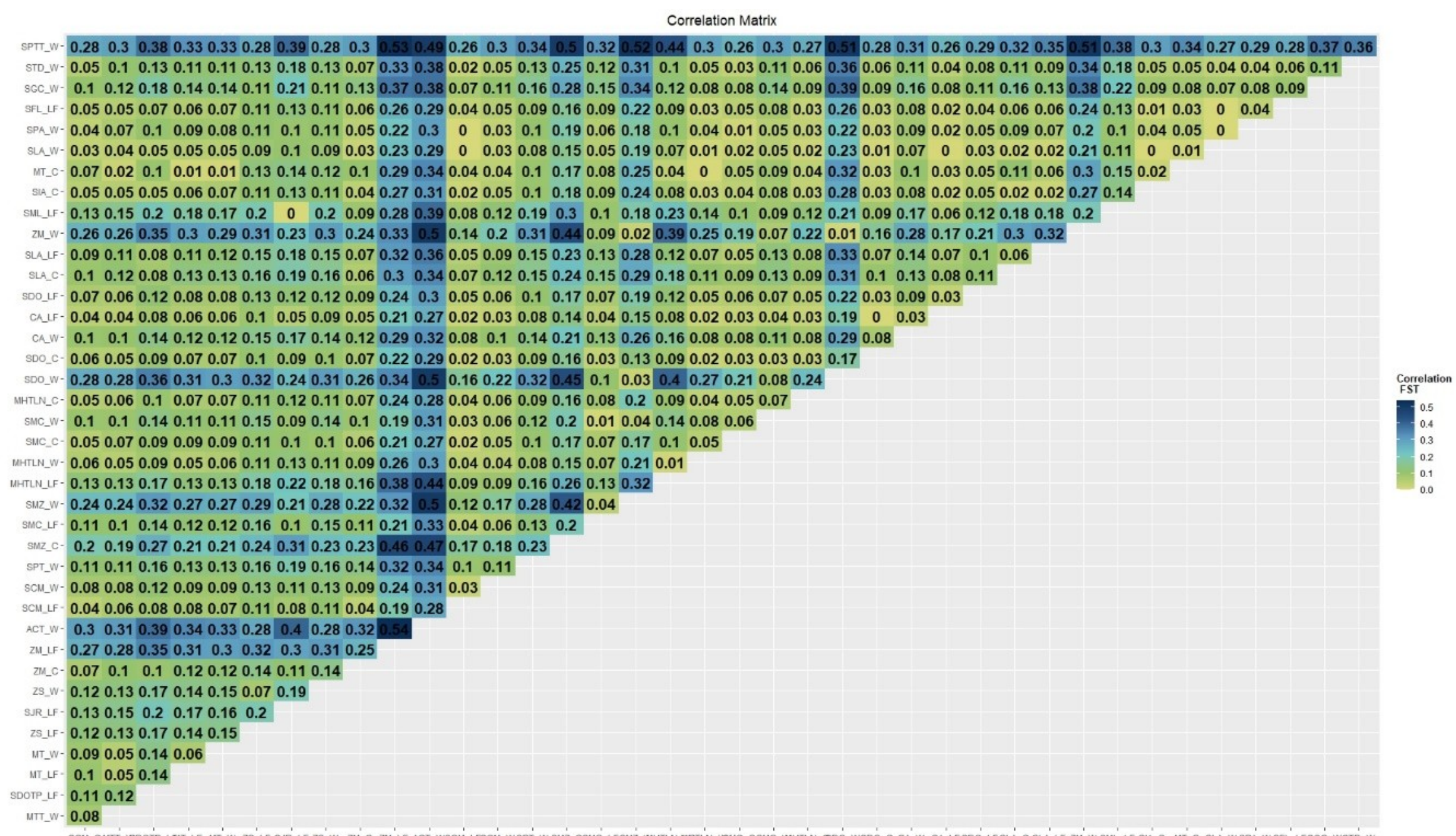

Figure S5. Pairwise F_{ST} matrix among sampled sites locations of *Agave karwinskii* collected across Puebla and Oaxaca.

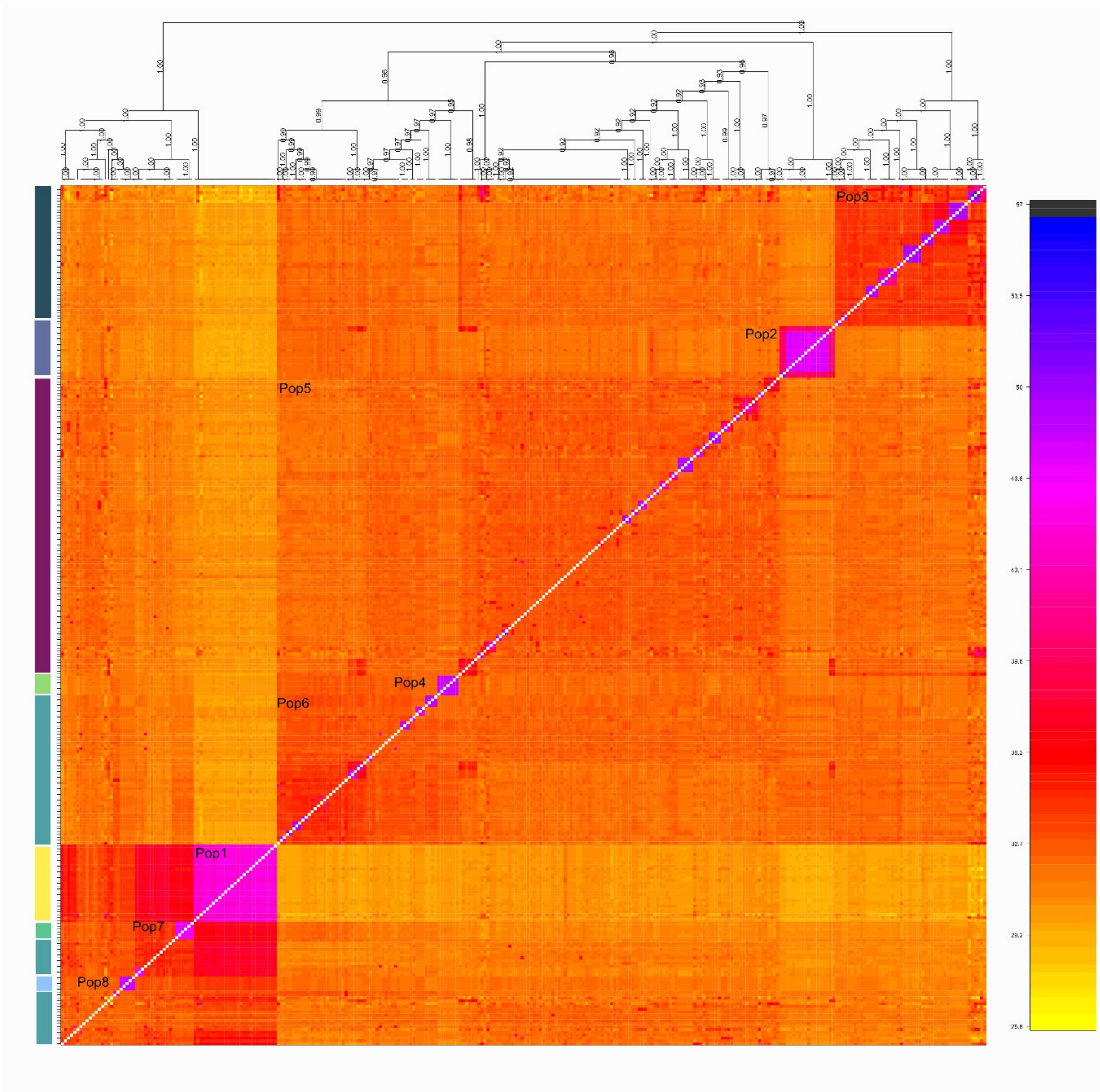

Figure S6. Results of the fineRADstructure analysis of genomic data from 300 individuals of *Agave karwinskii*, based on 9,578 SNPs. The heatmap represents pairwise co-ancestry variation among individuals, according to the color scale on the right, ranging from low relatedness (yellow) to high relatedness (blue/black). Colors correspond to the genetic groups (Pop, see text) identified using UPGMA clustering based on the proportion of shared loci.

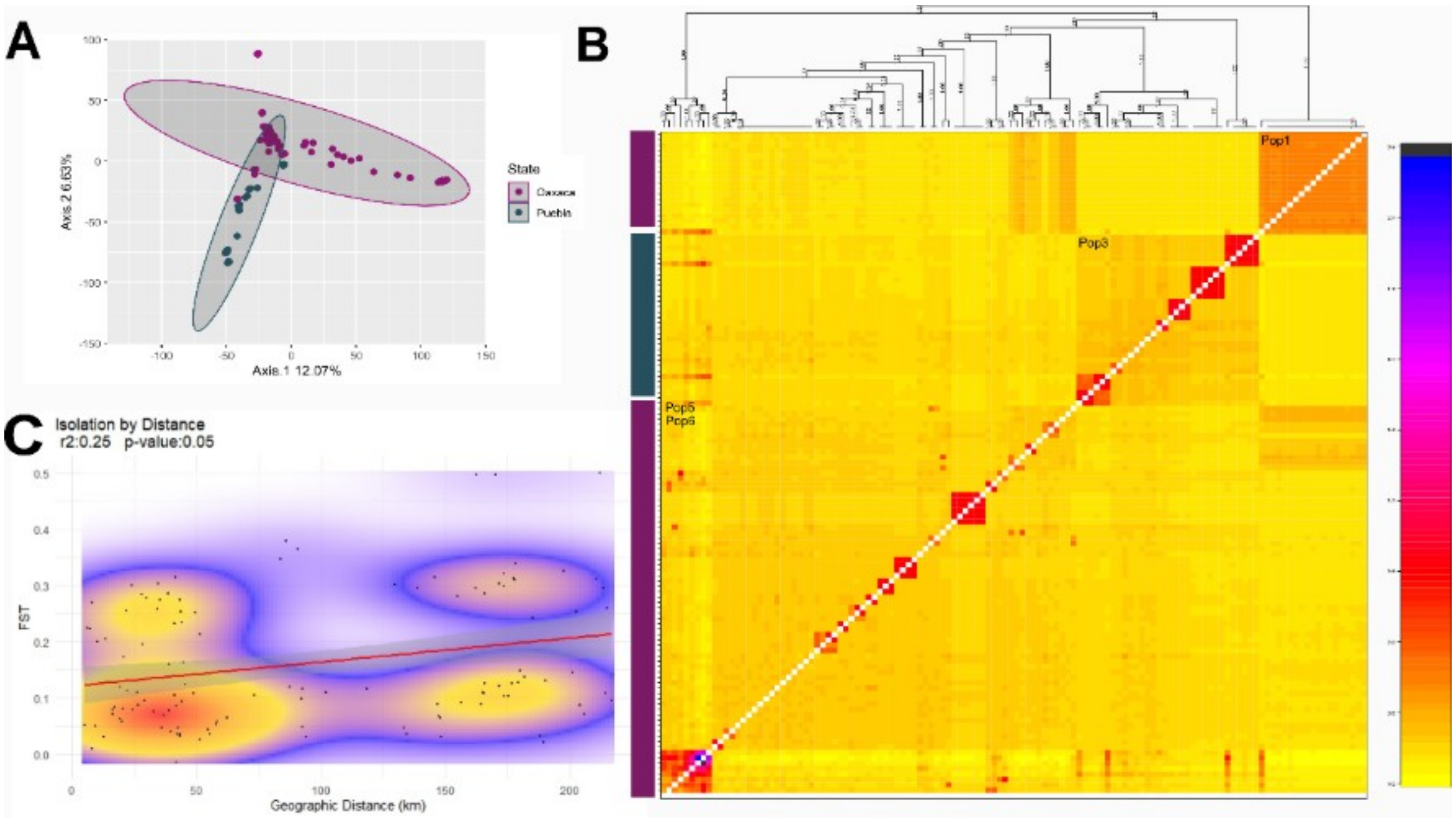

Figure S7. Structure analyses of 120 wild individuals of *Agave karwinskii* based on 10,632 SNPs. Principal Component Analysis (PCA) showing genetic differentiation consistent with the geographic distribution of individuals from Oaxaca and Puebla (A). fineRADstructure coancestry heatmap showing pairwise relatedness among individuals, where color intensity ranges from low (yellow) to high (blue/black). Sample colors indicate state of origin: Puebla (blue) and Oaxaca (purple) (B). Isolation by distance (IBD) pattern showing the relationship between pairwise F_{ST} and geographic distance (km). Black dots represent sampling sites, the red line indicates the regression trend, and colored ellipses highlight the main geographic groups.

Supplementary Tables

Table S1. Site information for the populations of *Agave karwinskii* collected across the states of Puebla and Oaxaca. Number of individuals used for genomic analysis (N). Samples were collected between 2022 and 2023.

ID	State	Management	Latitude	Longitude	Site name	N
1	Puebla	Wild	18.23916	-97.54888	ACT_W	6
2	Oaxaca	Live fence	16.5202	-96.56227	CA_LF	12
3	Oaxaca	Wild	16.522	-96.57064	CA_W	14
4	Oaxaca	Cultivated	16.36589	-96.52852	MHTLN_C	13
5	Oaxaca	Live fence	16.35532	-96.6091	MHTLN_LF	6
6	Oaxaca	Wild	16.35532	-96.6091	MHTLN_W	6
7	Oaxaca	Cultivated	16.92745	-96.3735	MT_C	5
8	Oaxaca	Live fence	16.921325	-96.36250556	MT_LF	4
9	Oaxaca	Wild	16.92152222	-96.35203333	MT_W	5
10	Oaxaca	Wild	16.89463	-96.39392	MTT_W	7
11	Oaxaca	Cultivated	16.7506175	-96.6170197	SCM_C	11
12	Oaxaca	Live fence	16.78075	-96.60774	SCM_LF	13
13	Oaxaca	Wild	16.78075	-96.60774	SCM_W	5
14	Oaxaca	Cultivated	16.78315	-96.66942	SDO_C	5
15	Oaxaca	Live fence	16.7496511	-96.660428	SDO_LF	10
16	Oaxaca	Wild	16.78315	-96.66942	SDO_W	5
17	Oaxaca	Live fence	16.80641	-96.39257	SDOTP_LF	3
18	Oaxaca	Live fence	16.4083333	-96.5525	SFL_LF	7
19	Oaxaca	Wild	18.15941	-97.14297	SGC_W	3
20	Oaxaca	Cultivated	16.33462	-96.49077	SIA_C	4
21	Oaxaca	Live fence	16.891111	-96.154999	SJR_LF	4
22	Oaxaca	Cultivated	16.36589	-96.52852	SLA_C	16
23	Oaxaca	Live fence	16.38771	-96.49702	SLA_LF	4
24	Oaxaca	Wild	16.36589	-96.52852	SLA_W	4
25	Oaxaca	Cultivated	16.64531	-96.76512	SMC_C	21
26	Oaxaca	Live fence	16.64621	-96.75634	SMC_LF	10
27	Oaxaca	Wild	16.64621	-96.75634	SMC_W	19
28	Oaxaca	Live fence	16.61296	-96.85271	SML_LF	5
29	Oaxaca	Cultivated	16.47457	-96.33757	SMZ_C	5
30	Oaxaca	Wild	16.48387	-96.35662	SMZ_W	3
31	Oaxaca	Wild	16.73534	-96.72568	SPA_W	4
32	Oaxaca	Wild	16.66624	-96.30201	SPT_W	6
33	Puebla	Wild	18.2716	-97.3113	SPTT_W	4
34	Oaxaca	Wild	17.65377	-96.91832	STD_W	3
35	Oaxaca	Cultivated	16.829536	-96.731605	ZM_C	6
36	Oaxaca	Live fence	16.829536	-96.731605	ZM_LF	6
37	Oaxaca	Wild	16.66430	-96.85360	ZM_W	3
38	Puebla	Live fence	18.315	-97.486	ZS_LF	11
39	Puebla	Wild	18.30399	-97.503	ZS_W	22

Table S2. Environmental and edaphological layers included in the genotype–environment association analysis using the RDA approach.

Abbreviation	Layer description
bdod_30	Bulk density of fine earth fraction at 15–30 cm
bdod_15	Bulk density of fine earth fraction at 5–15 cm
cfvo_30	Volumetric fraction of coarse fragments (> 2 mm) at 15–30 cm
sand_30	Sand content (> 0.05 mm) in fine earth at 15–30 cm
silt_30	Silt (0.002–0.05 mm) content in fine earth at 15-30 cm
soc_5	Soil organic carbon in fine earth at 5 cm
soc_30	Soil organic carbon in fine earth at 15–30 cm
AMT	Annual Mean Temperature
MDR	Mean Diurnal Range (Mean of monthly max temp – min temp)
PDM	Precipitation of the Driest Month
PWQ	Precipitation of the Wettest Quarter
PPDQ	Precipitation of the Driest Quarter
PPWQ	Precipitation of the Warmest Quarter
PPCQ	Precipitation of the Coldest Quarter

Table S3. Measures of genetic diversity and inbreeding in *Agave karwinskii* populations under different management regimes. Population abbreviations correspond to the names listed in Table S1, followed by the management type: wild (W), live fence (LF), and cultivated (C). Measures include observed (*Ho*) and expected heterozygosity (*Hs*); multilocus heterozygosity (*MLH*); standardized multilocus heterozygosity (*sMLH*); Wright's inbreeding coefficient (F_{IS}); inbreeding coefficient *f*; and the *Fhat3* index.

Site name	N	*Ho*	*Hs*	*MLH*	*sMLH*	F_{IS}	*f*	*fhat3*
ACT_W	6	0.2353	0.1240	0.2314	0.9945	-0.8499	0.0901	0.0868
CA_LF	12	0.2524	0.2599	0.2485	1.0699	0.0266	0.0206	0.0652
CA_W	14	0.2530	0.2253	0.2533	1.0901	-0.0743	0.0026	0.0072
MHTLN_C	13	0.2287	0.2406	0.2191	0.9425	0.0396	0.1371	0.1273
MHTLN_LF	6	0.1087	0.2237	0.0869	0.3778	0.3655	0.6543	0.6220
MHTLN_W	6	0.1765	0.2439	0.1543	0.6646	0.2013	0.3916	0.4043
MT_C	5	0.1720	0.2458	0.1140	0.4920	0.2030	0.5503	0.5482
MT_LF	4	0.2134	0.2212	0.2131	0.9177	-0.0033	0.1597	0.1408
MT_W	5	0.2142	0.2172	0.2135	0.9192	-0.0207	0.1583	0.1202
MTT_W	7	0.2295	0.2277	0.2299	0.9892	-0.0221	0.0945	0.0914
SCM_C	11	0.2177	0.2262	0.2176	0.9369	0.0232	0.1425	0.1017
SCM_LF	13	0.2115	0.2471	0.1694	0.7292	0.1120	0.3322	0.3237
SCM_W	5	0.2741	0.2657	0.2727	1.1765	-0.0601	-0.0794	0.0798
SDO_C	5	0.2445	0.2542	0.2444	1.0507	0.0146	0.0386	0.0600
SDO_LF	10	0.2233	0.2335	0.2198	0.9468	0.0425	0.1334	0.1145
SDO_W	5	0.3074	0.1559	0.3063	1.3184	-0.9596	-0.2060	-0.1541
SDOTP_LF	3	0.2459	0.2165	0.2458	1.0555	-0.1729	0.0331	0.0577

SFL_LF	7	0.2307	0.2401	0.2307	0.9928	0.0240	0.0911	0.0763
SGC_W	3	0.1827	0.2073	0.1470	0.6332	0.0061	0.4197	0.3857
SIA_C	4	0.2317	0.2438	0.1949	0.8391	0.0058	0.2320	0.2121
SJR_LF	4	0.2274	0.2271	0.2267	0.9765	-0.0350	0.1063	0.0829
SLA_C	16	0.2469	0.2185	0.2402	1.0340	-0.0705	0.0533	0.0373
SLA_LF	4	0.2093	0.2350	0.1968	0.8470	0.0302	0.2233	0.2487
SLA_W	4	0.2438	0.2543	0.2440	1.0497	0.0105	0.0393	0.0342
SMC_C	21	0.2349	0.2490	0.2148	0.9250	0.0612	0.1526	0.1678
SMC_LF	10	0.2218	0.2345	0.1837	0.7909	0.0318	0.2757	0.2757
SMC_S	19	0.2578	0.2401	0.2556	1.0999	-0.0288	-0.0063	0.0207
SML_LF	4	0.2606	0.2090	0.2602	1.1202	-0.2707	-0.0252	-0.0135
SMZ_C	3	0.2165	0.1479	0.1720	0.7394	-0.4899	0.3229	0.3260
SMZ_W	3	0.2626	0.1557	0.2455	1.0580	-0.6450	0.0324	0.0567
SPA_W	4	0.2521	0.2500	0.2590	1.1140	-0.0324	-0.0209	0.0177
SPT_W	6	0.2133	0.2111	0.2096	0.9007	-0.0333	0.1750	0.1500
SPTT_W	4	0.2275	0.1147	0.2272	0.9776	-0.9731	0.1051	0.0835
STD_W	3	0.2551	0.2394	0.2405	1.0362	-0.1267	0.0498	0.1096
ZM_C	8	0.2457	0.2305	0.2458	1.0581	-0.0710	0.0316	0.0229
ZM_LF	3	0.2066	0.1310	0.2012	0.8657	-0.5671	0.2079	0.1943
ZM_W	4	0.2954	0.1524	0.2772	1.1950	-0.9301	-0.0932	-0.0653
ZS_LF	14	0.2096	0.2190	0.1788	0.7701	0.0613	0.2951	0.2836
ZS_W	22	0.2159	0.2152	0.1941	0.8347	0.0194	0.2359	0.2304

Table S4. Functional annotation and characteristics of consensus candidate SNPs identied as putatively under selection in *Agave karwinskii*.

Protein name	Gene	Function	Reference
Pentatricopeptide repeat-containing protein	J5N97_024944	Profound effects on organelle biogenesis and function, and consequently on photosynthesis, respiration, plant development, and environmental responses.	Barkan and Small, (2014)
DUF642 domain-containing protein	A4U43_C05F18080	Possible role of this protein family during the germination process.	Zúñiga-Sánchez *et al.*, (2014)
Proteasome subunit alpha type (UPS)	A4U43_C07F36730	Involved in nearly all steps of plant defence mechanisms.	Dielen *et al.*, (2010)
AP2/ERF domain-containing protein	A0A5P1F8D9_ASPOF	Plant morphogenesis, responses to various types of stress, hormonal signal transduction, and metabolite regulation.	Feng *et al.*, (2020)

C2H2-type domain-containing protein	A4U43_C09F9340	Plays vital roles in shaping many aspects of plant growth and environmental adaptation.	Jiao *et al.*, (2020)
Allene oxide synthase	A0A804IGQ5_MUSAM	AOS produces most non-volatile oxylipins in plants, including the defence hormone jasmonate.	Farmer and Goossens, (2019)
Protein-serine/ threonine phosphatase, 3.1.3.16 (2)	E1A91_D05G000900v1	Oxidative stress signalling in plants.	Máthé *et al.*, (2019)
V-type proton ATPase subunit e1	LOC109728794	Improves cation exchange capacity and enhances tolerance to salt stress.	Futai *et al.*, (2019)
Activator of 90 kDa heat shock protein ATPase homolog	LOC105041894	Proteins that play a key role in heat stress responses in living cells.	Xu *et al.*, (2012)
Ultraviolet-B-repressible protein	COCNU_05G008870	Increases upon plant exposure to UV radiation, enhancing the production of reactive oxygen species (ROS).	Borges *et al.*, (2012)
Glycosyltransferase family 47 IRX10	A4U43_C09F15820	A plant-dominated enzyme family likely involved in cell wall biosynthesis and localized to Golgi vesicles.	Geshi *et al.*, (2010)
Protein-serine/ threonine	LOC105058604	Implicated in various fundamental processes in plant growth and development.	Mahipal (2017)
Indole-3-acetic acid-amido synthetase GH3.5	MUK42_13099	Involved in drought adaptation; induced by drought, abscisic acid (ABA), and high salinity.	Xiaodong Liu *et al.*, (2016)
Glycosyltransferase STELLO2	A4U43_C02F14520	Closely associated with cellulose biosynthesis through the regulation of cellulose synthase complex assembly and trafficking.	Sampaio *et al.*, (2020)
Scarecrow-like protein 6	J5N97_029846	Key regulators of root growth and asymmetric cell divisions separating ground tissue into endodermis and cortex.	Koizumi and Gallagher, (2013)

Table S5. Contemporary effective population size (N_e) estimates for *Agave karwinskii* genetic groups (Pop). Number of samples analyzed (N); approximation of the census size according to the direct observation in the field (N_c): Confidence interval of 95% is shown in parentheses. Excluding singletons only (ES).

Pop	N	N_c	N_e in NeEstimator			N_e in Colony2		
			ES SNP: 21, 371	MAF: 0.02 SNP: 5, 931	MAF: 0.05 SNP: 3, 436	ES SNP: 21, 371	MAF 0.02 SNP: 5, 931	MAF 0.05 SNP: 3, 436
1	27	280	1.4 (1.0-2.0)	1.2 (0.8-1.8)	1.0 (1.6-1.6)	3 (2-infinite)	5 (2-20)	5 (2-20)
2	16	12,000	0.5 (0.4-0.7)	0.5 (0.4-0.6)	0.5 (0.4-0.6)	4 (2-15)	3 (2-infinite)	3 (2-infinite)
3	57	975	3.7 (2.9-8.6)	3.6 (2.6-7.1)	5.5 (3.3-9.1)	32 (19-52)	36 (22-61)	41 (27-67)
4	7	20	-	-	-	2 (2-infinite)	2 (2-infinite)	2 (2-infinite)
5	141	5,650	17.5 (12.5-22.7)	40.6 (30.8-66.6)	46.6 (34.3-62.1)	139 (106-179)	123 (95-159)	118 (88-131)
6	41	1,150	13.4 (7.9-37.4)	16.8 (9.9-66.7)	17.6 (11.4-65.6)	89 (63-130)	78 (52-128)	68 (43-111)
7	5	30	-	-	-	-	2 (2-infinite)	2 (2-infinite)
8	6	30	-	-	-	-	2 (2-infinite)	2 (2-infinite)

Table S6. Results of the two-way ANOVA testing the effects of management type and estimation program on log-transformed N_e estimates. Sum of squares (Sum sq); degrees of freedom (df); probability value associated with the F statistic (Pr (>F)).

Source of Variation	Sum Sq	df	Mean Sq	F value	Pr (>F)
Management Type	17.58	3	5.86	9.04	0.001
Program	20.25	1	20.25	31.25	< 0.001
Management: Program (Interaction)	3.32	3	1.11	1.71	0.205
Residuals	9.72	15	0.65		

Table S7. Contemporary N_e estimates for *Agave karwinskii* genetic components by management. Genetic group (Pop); number of samples analyzed (N); approximation of the census size according to the direct observation in the field (N_c); confidence interval of 95% is shown in parentheses; excluding singletons only (ES).

Pop	Type of management	N	N_c	N_e in NeEstimator			N_e in Colony2		
				ES	MAF: 0.02	MAF: 0.05	ES	MAF: 0.02	MAF: 0.05
1	*Ex situ* promotion	14	130	3 (0.3-infinite)	4.6 (0.2-infinite)	4.4 (1.4-infinite)	3 (2-infinite)	3 (2-infinite)	3 (2-infinite)
	Wild	13	150	3 (1.2-	3.2 (1.2-	3.2 (1.1-	4 (2-17)	4 (2-15)	4 (2-12)

				112.9)	infinite)	infinite)			
2	Cultivated by vegetative propagation	16	12000	0.6 (0.4-0.7)	3 (2-infinite)	0.5 (0.4-0.6)	3 (2-infinite)	3 (2-infinite)	3 (2-infinite)
3	*Ex situ* promotion	19	75	1.3 (0.6-5.3)	1.1 (0.6-2.6)	1.1 (0.5-1.9)	22 (10-45)	24 (12-47)	26 (15-53)
	Wild	38	900	3.5 (2.4-11.2)	2.3 (1.5-4.4)	2.2 (1.5-3.3)	23 (13-45)	25 (15-46)	27 (17-47)
	Cultivated by vegetative propagation	20	700	8.3 (2.9-137.9)	5 (1.4-17.8)	4.6 (2.0-27.3)	26 (14-46)	26 (15-51)	29 (16-57)
5	*Ex situ* promotion	60	3500	20.9 (3.8-90.3)	33.9 (19.8-77.7)	37.7 (20.9-80.4)	156 (107-224)	154 (109-226)	154 (113-229)
	Tolerated	25	350	23.2 (8.1-32.2)	17.7 (7.5-46.1)	15.3 (7.6-33.5)	78 (47-150)	75 (44-145)	60 (35-116)
	Wild	25	600	10.6 (9.2-infinite)	9.2 (3-24.2)	8.1 (2.7-25.3)	48 (27-85)	46 (27-84)	46 (27-84)
6	*Ex situ* promotion	20	200	15.3 (7.2-infinite)	10.7 (2.8-49.3)	9.5 (2.7-93.6)	42.5 (23-84)	42 (24-84)	42 (24-84)
	Cultivated by vegetative propagation	9	750	1.9 (0.6-infinite)	1.6 (0.4-7.9)	1.5 (0.5-10.2)	18.5 (7-52)	18 (8-52)	18 (8-52)

Supplementary references

This list includes the scientific sources associated with the UniProt entries that matched our BLASTx searches of the consensus sequences containing candidate SNPs (Table S4).

Barkan A, Small I. 2014. Pentatricopeptide repeat proteins in plants. *Annual Review of Plant Biology*, 65: 415–442. https://doi.org/10.1146/annurev-arplant-050213-040159

Borges A, Melotto M, Tsai SM, Caldas DGG. 2012. Changes in spatial and temporal gene expression during incompatible interaction between common bean and anthracnose pathogen. *Journal of Plant Physiology*, 169(12): 1216–1220. https://doi.org/10.1016/j.jplph.2012.04.003

Dielen AS, Badaoui S, Candresse T, German-Retana S. 2010. The ubiquitin/26S proteasome system in plant–pathogen interactions: a never-ending hide-and-seek game. *Molecular plant pathology*, 11(2): 293-308.https://doi.org/10.1111/j.1364-3703.2009.00596.x

Farmer EE, Goossens A. 2019. Jasmonates: What ALLENE OXIDE SYNTHASE does for plants. *Journal of Experimental Botany*, 70(13): 3373–3378. https://doi.org/10.1093/jxb/erz254

Feng K, Hou XL, Xing GM, *et al*., Xiong AS. 2020. Advances in AP2/ERF super-family transcription factors in plants. *Critical Reviews in Biotechnology*, 40(6): 750–776. https://doi.org/10.1080/07388551.2020.1768509

Futai M, Sun-Wada GH, Wada Y, Matsumoto N, Nakanishi-Matsui M. 2019. Vacuolar-type ATPase: A proton pump to lysosomal trafficking. *Proceedings of the Japan Academy Series B*, 95(6), 261–277.https://doi.org/10.2183/pjab.95.018

Geshi N, Harholt J, Sakuragi Y, Jensen JK, Scheller HV. 2010. Glycosyltransferases of the GT47 family. *Annual Plant Reviews*: Plant Polysaccharides, Biosynthesis and Bioengineering, 41, 265-283.https://doi.org/10.1002/9781444391015.ch10

Jiao Z, Wang L, Du H, et al., Ge, L. 2020. Genome-wide study of C2H2 zinc finger gene family in Medicago truncatula. BMC *Plant Biology*, 20: 1-17.https://doi.org/10.1186/s12870-020-02619-6

Koizumi K, Gallagher KL 2013. Identification of SHRUBBY, a SHORT-ROOT and SCARECROW interacting protein that controls root growth and radial patterning. *Development*, 140(6), 1292-1300.https://doi.org/10.1242/dev.090761

Máthé C, Garda T, Freytag C, M-Hamvas M. 2019. The role of serine-threonine protein phosphatase PP2A in plant oxidative stress signaling—facts and hypotheses. *International Journal of Molecular Sciences*, 20(12), 3028.https://doi.org/10.3390/ijms20123028

Sampaio M, Neves J, Pereira S, Pissarra J, Pereira C. 2020. Dissecting Plant Specific Insert Interaction Networks. In *Biology and Life Sciences Forum* (Vol. 4, No. 1, p. 65). MDPI.https://doi.org/10.3390/IECPS2020-08870

Xiaodong Liu, Ruozhong Wang, Binbin Jiao, Peihong Dai, Yue Li. 2016. Indole Acetic Acid-Amido Synthetase GH3-6 Negatively Regulates Response to Drought and Salt in Arabidopsis[J]. *Chinese Bulletin of Botany*, 51(5): 586-593.DOI: 10.11983/CBB15223

Xu ZS, Li ZY, Chen Y, Chen M, Li LC, Ma YZ. 2012. Heat shock protein 90 in plants: molecular mechanisms and roles in stress responses. *International journal of molecular sciences*, 13(12), 15706-15723.https://doi.org/10.3390/ijms131215706

Mahipal. 2017. Functional Characterization of C3HC4-type RING Zinc Finger Protein Gene from Hot Pepper. PhD Thesis, Seoul National University, South Korea.

Zúñiga-Sánchez E, Soriano D, Martínez-Barajas E, Orozco-Segovia A, Gamboa-deBuen A. 2014. BIIDXI, the At4g32460 DUF642 gene, is involved in pectin methyl esterase regulation during Arabidopsis thaliana seed germination and plant development. *BMC Plant Biology*, 14, 1-13. https://doi.org/10.1186/s12870-014-0338-8
